# A systematic review of barriers to and facilitators of the use of evidence by policymakers

**DOI:** 10.1186/1472-6963-14-2

**Published:** 2014-01-03

**Authors:** Kathryn Oliver, Simon Innvar, Theo Lorenc, Jenny Woodman, James Thomas

**Affiliations:** 1 School of Social Sciences, University of Manchester, Bridgeford Street, M13 9PL Manchester, UK; 2 Faculty of Social Sciences, Oslo University College, P.B. 4, St. Olavs Plass, NO-0130 Oslo, Norway; 3 Department of Science, Technology, Engineering, and Public Policy (UCL STEaPP), University College London, 66-72 Gower Street, London WC1E 6EA, UK; 4 MRC Centre of Epidemiology for Child Health, Institute of Child Health, London WC1N 1EH, UK; 5 University of London, Institute of Education, 20 Bedford Way, London WC1H 0AL, UK

## Abstract

**Background:**

The gap between research and practice or policy is often described as a problem. To identify new barriers of and facilitators to the use of evidence by policymakers, and assess the state of research in this area, we updated a systematic review.

**Methods:**

Systematic review. We searched online databases including Medline, Embase, SocSci Abstracts, CDS, DARE, Psychlit, Cochrane Library, NHSEED, HTA, PAIS, IBSS (Search dates: July 2000 - September 2012). Studies were included if they were primary research or systematic reviews about factors affecting the use of evidence in policy. Studies were coded to extract data on methods, topic, focus, results and population.

**Results:**

145 new studies were identified, of which over half were published after 2010. Thirteen systematic reviews were included. Compared with the original review, a much wider range of policy topics was found. Although still primarily in the health field, studies were also drawn from criminal justice, traffic policy, drug policy, and partnership working. The most frequently reported barriers to evidence uptake were poor access to good quality relevant research, and lack of timely research output. The most frequently reported facilitators were collaboration between researchers and policymakers, and improved relationships and skills. There is an increasing amount of research into new models of knowledge transfer, and evaluations of interventions such as knowledge brokerage.

**Conclusions:**

Timely access to good quality and relevant research evidence, collaborations with policymakers and relationship- and skills-building with policymakers are reported to be the most important factors in influencing the use of evidence. Although investigations into the use of evidence have spread beyond the health field and into more countries, the main barriers and facilitators remained the same as in the earlier review. Few studies provide clear definitions of policy, evidence or policymaker. Nor are empirical data about policy processes or implementation of policy widely available. It is therefore difficult to describe the role of evidence and other factors influencing policy. Future research and policy priorities should aim to illuminate these concepts and processes, target the factors identified in this review, and consider new methods of overcoming the barriers described.

## Background

Despite an increasing body of research on the uptake and impact of research on policy, and encouragement for policymaking to be evidence-informed [1], research often struggles to identify a policy audience. The research-policy gap’ is the subject of much commentary and research activity [2-4]. Interventions to bridge this gap are the focus of recent systematic reviews [5-7]. To ensure these interventions are appropriately designed and effective, it is important that they address genuine barriers to research uptake, and utilise facilitators which are likely to affect research uptake.

It is now well recognized that policy is determined as much by the decision-making context (and other influences) as by research evidence [8,9]. Policymakers’ perceptions form an important part of this story, but not the whole. Innvaer [10] aimed to review studies about the health sector, but the influence of the evidence-based policy movement is now recognized to be important across many policy areas. In the UK, with the creation of Clinical Commissioning Groups, Health and Well-Being Boards, and private providers moving into areas traditionally occupied by the NHS, a broader range of policymakers are becoming potential evidence-users’ than ever. Researchers need to take stock of what we know about evidence-based policy, what we don’t know, and what can be done to assist these users.

The last systematic review looking at policymakers’ perceptions about the barriers to, and facilitators of research use was Innvaer [10]. The findings from this review were corroborated by later research [11,12], but no systematic update has yet been undertaken. In addition to updating this review in the area of policymakers’ perceptions of barriers and facilitators to use of evidence in policy, we also wished to include perceptions from other stakeholder groups than policymakers, such as researchers, managers, and other research users. Furthermore, it may be possible to identify factors affecting research use without relying on the perceptions of research participants – for example, ethnographic studies may produce observational data about knowledge exchange. In addition, we acknowledge that interest in using evidence to inform policy has spread beyond the health sector. Therefore, we aimed to update Innvaer [10] to include studies identifying all barriers and facilitators of the use of evidence in all policy fields.

This review aimed to update and expand Innvaer [10], and broaden the scope of the review to:

• Identify factors which act as barriers to and facilitators of the use of evidence in public policy, including factors perceived by different stakeholder groups.

• Describe the focus, methods, populations, and findings of the new evidence in this area.

Because this review has a larger scope that Innvaer [10], caution must be used in drawing direct comparisons; discussed further in the results.

## Methods

A protocol for the review was developed and sent to an advisory group of senior academics (available from KO) in order to ensure that the methods and search strategies were exhaustive.

To be included, studies had to be:

• Primary research (any study design) or systematic reviews categorising, describing or explaining how evidence is used in policymaking. Intervention studies were included.

• About policy (defined as decisions made by a state organisation, or a group of state organisations, at a national, regional or conurbation level). Studies of clinical decision-making for individual patients, or protocols for single clinical sites were excluded.

• About barriers or facilitators to the use of evidence (relational, organisational, factors related to researchers, policymakers, policy or research directly, or others).

We did not exclude any studies on the basis of population. These criteria are therefore broader than those for Innvaer [10] by including all study designs, all populations and all policy areas.

The following electronic databases were searched using adapted search strings from Innvaer [10] from July 2000 (the cut-off point for the earlier review) - September 2012: Medline, Embase, SocSci Abstracts, CDS, DARE, Psychlit, Cochrane Library, NHSEED, HTA, PAIS, IBSS. Searches combined policy’ terms with utilisation/use’ terms in the first instance. The full search strategy is available from the corresponding author on request; sample search available here (Additional file 1). Authors in the field were contacted and key websites were hand-searched. In order to pick up a range of study designs and theoretical papers a methodological filter was not applied.

All studies were screened initially on title and abstract. 100 studies were double screened to ensure consistency, and revisions were made to definitions and criteria accordingly. Relevant studies were retrieved and screened on full text by one reviewer.

Studies were stored, screened and keyworded using the EPPI Reviewer software [13]. Data were extracted on study characteristics, sampling and recruitment, theoretical framework, methods, and results, with all studies being coded by one reviewer, and two reviewers coding 10-25% each (67 studies were double-coded in total). Because we were not aiming to determine the size of an effect, but instead to describe a body of literature, no risk of bias assessment was made. Quality appraisal in this case would have made no difference to this systematic descriptive synthesis.

Studies were keyworded using a data extraction tool which collected information on study characteristics, topic and focus, and theoretical background. Factors which affected evidence use were coded as barriers or facilitators against a pre-defined list of factors, which was iteratively updated as new factors were identified. All studies were therefore coded at least twice, once with the initial tool, and once with the finalised list of factors.

## Results

**Figure 1:**
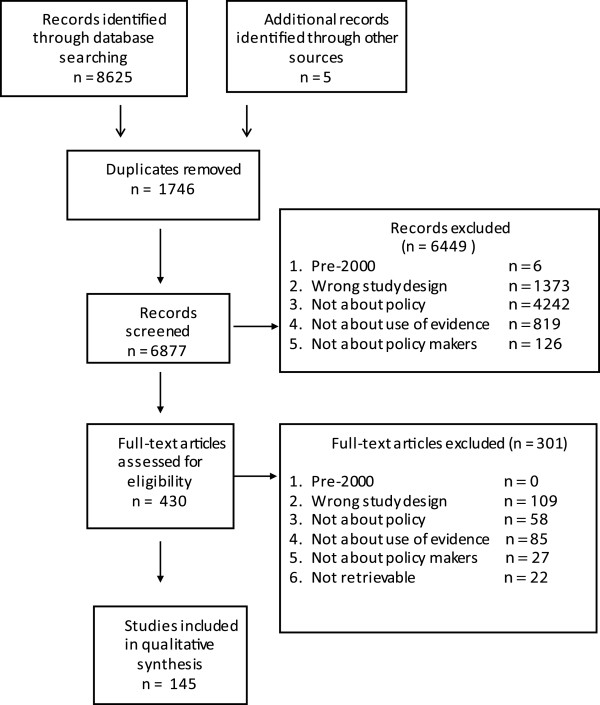
PRISMA flowchart detailing flow of studies through the review.

6879 unique records were retrieved, of which 430 were screened on full text. 145 studies were included on full text, of which half were published between September 2010 and September 2012. Figure 1 describes the flow of studies through searching and screening for inclusion.

### Characteristics of included studies

For a full description of the included studies, see Additional file 2. Studies were undertaken in a wide range of countries (145 studies in over 59 countries,). A significant proportion (n = 33, 23%) were from low- and middle-income countries in regions such as sub-Saharan Africa and Central America (n = 32), and several were conducted in Middle-Eastern states (n = 4).

Eleven studies used observational (ethnographic) methods to collect data, and 37 used documentary analysis. However, these represent less than a quarter of included studies, the majority of which were or included semi-structured interviews (n = 79), or included a survey (n = 44). Twelve studies were longitudinal while the rest were cross-sectional. Thirteen systematic reviews and fifty-three case studies were included.

Most studies reported perceptions or experiences of respondents (n = 109; n = 64 respectively), rather than documentary proof or observational results about the use of evidence in policy (n = 14; n = 11 respectively). Evidence’ was defined in 121 studies. Where it was possible to identify what kinds of evidence were being discussed, most focused on the use of research evidence (n = 90) with 33 focusing specifically on systematic reviews. However, 59 studies looked at the use of non-formal evidence, which included local data, surveillance data, personal experience, clinical expertise, or other informal knowledge.

**Figure 2:**
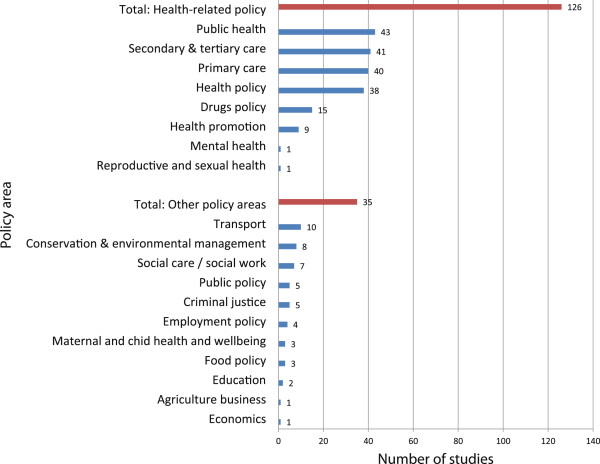
Policy focus of study.

The context of the study was usually non-specific, referring to general policy (n = 84) or practice (n = 37). Changes to specific policy areas or policies were explored in 22 and 14 studies respectively, and information/evidence diffusion in 13. Some studies explicitly set out to look at uptake or adoption of research (n = 41), and others described interventions aiming to increase uptake [14], or the context after a specific piece of research or policy (such as after the introduction of the 1999 White Paper “Saving Lives: our healthier nation” [15,16]. The vast majority of studies were conducted in health or health-related fields. Most new evidence in the area focused on the health sector, but research was also conducted in areas including traffic [17,18], criminal justice [19-23], drugs policy [22,24-37], and environmental conservation [20,22,38-42] (see Figure 2).

### Who are these studies written by and for?

137 study reports were written by researchers or people with academic affiliations, with clinical researchers co-authoring a proportion of these (n = 57). Policymakers were credited as authors in 3 studies, [25,39,43] and one of those was a governmental report.

**Figure 3:**
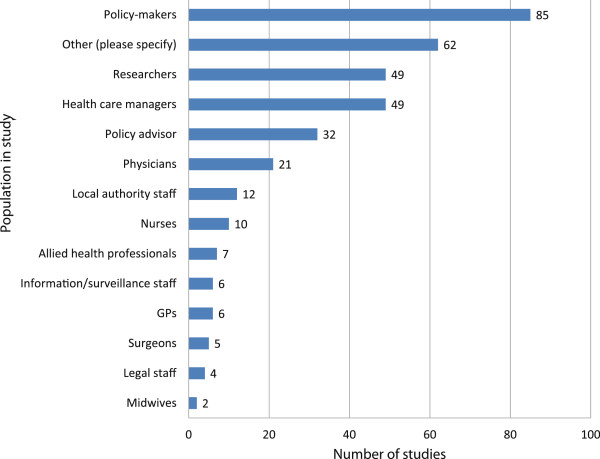
Sample population.

The population samples themselves were predominantly policymakers or advisors (n = 86, n = 32 respectively), health care managers (n = 49), or researchers (49), although many other groups were also included (see Figure 3). Where researchers were included in the study population (n = 49), they often outnumbered the policy and practice participants. Other participants included commissioners, health economists, third sector workers, patients, industry and business representatives, and justice and criminal workers. Because it was not always clear who had been involved and what their roles were, it was not possible to give numbers for all these groups. Also included in this other’ category (n = 62) were all documents analysed.

### What factors affect use of evidence?

**Figure 4:**
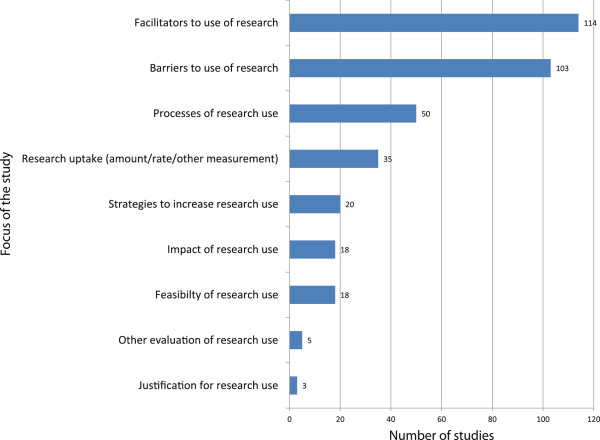
Main barriers and facilitators of the use of evidence by policymakers.

All studies reported either barriers, facilitators, or both, of the use of evidence. Studies also described processes of research use (n = 50), strategies and interventions to increase research use (n = 24), and assessments of the uptake of research (n = 33) (see Figure 4).

Studies reported a range of factors which acted as barriers and/or facilitators of evidence use. The most frequently reported barriers were the lack of availability to research, lack of relevant research, having no time or opportunity to use research evidence, policymakers’ and other users not being skilled in research methods, and costs (see Table 1). The most frequently reported facilitators also included access to and improved dissemination of research, and existence of and access to relevant research. Collaboration and relationships between policymakers and research staff were all reported as important factors.

**Table 1:** Most frequently reported barriers and facilitators of the use of evidence (n = # studies in which factor reported)

**Top 5 barriers to use of evidence**	**Top 5 facilitators of evidence use**
• Availability and access to research/improved dissemination (n = 63)	• Availability and access to research/improved dissemination (n = 65)
• Clarity/relevance/reliability of research findings (n = 54)	• Collaboration (n = 49)
• Timing/opportunity (n = 42)	• Clarity/relevance/reliability of research findings (n = 46)
• Policymaker research skills (n = 26)	• Relationship with policymakers (n = 39)
• Costs (n = 25)	• Relationship with researchers/info staff (n = 37)

To interpret all the factors reported by included studies, the barriers and facilitators were categorised into themes depending on content: Organisations and resources’ , Contact and collaboration’ , Research and researcher characteristics’ , Policymaker characteristics’ , Policy characteristics’ , and Other’ (see Table 2). Below, we describe the main barriers and facilitators reported within each theme, and we give some supplementary information not mentioned in the table.

**Table 2:** Barriers and Facilitators categorised into themes (n = number of studies)

**Seen as barrier**		**Factor**	**Seen as facilitator**	
**85**	**Contact and collaboration**			**98**
	8	Collaboration	49	
	42	Timing/opportunity	24	
	8	Relationship with policymakers	39	
	8	Relationship with researchers/info staff	37	
	8	Contact with researchers/info staff	31	
	9	Contact with policymakers	30	
	2	Other	1	
92	**Organisation and resources**			**99**
	63	Availability and access to research/improved dissemination	65	
	25	Costs	11	
	3	Managerial support (practical)	22	
	11	Professional bodies	15	
	11	Material resources available	12	
	14	Staff or personnel resources	10	
	3	Managerial will	5	
	9	Staff turnover/continuity of employment	3	
	9	Other	9	
85	**Research and researcher characteristics**			**95**
	54	Clarity/relevance/reliability of research findings	46	
	18	Format of research findings	26	
	9	Importance of research findings	10	
	25	Other	32	
62	**Policymaker characteristics**			**69**
	26	Policymaker research skills	22	
	24	Policymaker research awareness	10	
	13	Political support (will)	21	
	4	Political support (practical)	12	
	12	Practitioner research skills	6	
	3	Practitioner research awareness	2	
	6	Other	11	
28		**Policy characteristics**		**33**
	6	Guidelines or policy statement	9	
	9	Importance of policy	11	
	5	Legal or legislative support	3	
	26	Other pressures on policy	5	
	4	Other	4	
10		**Other**		**1**
	9	Consumer-related barrier	0	
	1	Other	1	
105	**All factors (total)**			**124**

### Contact and relationships

Contact, collaboration and relationships are a major facilitator of evidence use, reported in over two thirds of all studies. Timing and opportunity was the most prominent barrier (n = 42) within this theme. Many studies also discussed the role of relationships, trust, and mutual respect. The serendipitous nature of the policy process was emphasised in some studies, which discussed the role of informal, unplanned contact in policy development and in finding evidence.

### Organisations and resources

Organisational factors such as lack of access to research, poor dissemination and costs were highly reported factors affecting the use of research. Other barriers were lack of managerial support, professional bodies, material and personnel resources, managerial will and staff turnover. Professional bodies were seen as barriers where useful guidelines were not available, or where they were perceived to be political or biased. In the case of the WHO, it was seen as unreliable, unsupportive, and with dubious claims to be evidence-based’ [31,44]. Other factors mentioned in connection with organisational and resource barriers included poor long term policy planning [45], inflexible and non-transparent policy processes [46,47] and in developing countries, lack of effective health care systems [24]. Leadership and authority were reported as facilitators, with emphasis on community leadership [48] and policy entrepreneurialism of policy champions [43,49].

Among the facilitators under the theme *Organisation and Resources*, availability, access and dissemination were considered important facilitators, as was managerial support (n = 22).

### Research and researcher characteristics

Characteristics of research evidence were widely reported as factors affecting uptake of research, with clarity, relevance and reliability of research findings reported as important factors. The format of research output was also an important factor in uptake. The importance of the research findings themselves was discussed in 19 studies, usually studies describing the uptake of health inequalities research. The quality and authoritativeness of research was clearly a factor in uptake, particularly where other evidence in the area was poor quality [50].

Emerging as a new stream of research, eleven studies evaluated or described knowledge broker roles or related concepts [6,35,37,51-57] with dedicated dissemination strategies evaluated in 7 studies and mentioned as a facilitator in 43. Incentives to use evidence and client demand for research evidence were described as facilitators in one study each [10,58].

Researchers themselves were described as factors affecting uptake of their research. Having a good understanding of the policy process and the context surrounding policy priorities was supportive of research uptake [17,18,59-61]. A barrier to uptake was identified where researchers were described as having different priorities from policymakers, with pressure to publish in peer-reviewed journals [27,62,63]. Researchers were valued more when it was clear they were non-partisan and producing unbiased results [40,57,64], and provision of expert advice was also reported as helpful.

### Policymaker characteristics

Policymakers’ characteristics were also reported to play a role in evidence uptake, with their research skills and awareness (or lack of) reported as a barrier in 34 studies. Some studies reported that policymakers’ beliefs about the utility of evidence-use was a major factor in evidence use (barrier: n = 2, facilitator: n = 3), and, in general, personal experiences, judgments, and values were reported as important factors in whether evidence was used. However, these findings were nearly all (91%) based on studies of perceptions, of which half were perceptions of researchers.

Some studies reported that left-leaning, younger and/or female policymakers were more likely to use research evidence [65,66]. Being more highly educated was reported as a barrier [67], but there was no consensus about the effect of being clinically trained [61,68].

### Policy characteristics

Perhaps surprisingly, legal support and the existence of guidelines for the use of evidence were scarcely reported as factors affecting uptake of evidence. The importance and complexity of the policy area was also discussed, especially in comparison with the relative simplicity of clinical problems.

However, competing pressures (economic, political, social, and cultural factors) were seen to impact on the policy process and hinder the development of evidence-based policy. Political pressures, finances, and competing priorities were all discussed (n = 12), with the media (n = 3) vested interest and pressure/lobby groups (n = 3) and unclear decision-making practices (n = 2) also reported as barriers.

### Other factors

One study which studied use of evidence in prisons reported potential security breaches from data loss as a potential barrier to evidence use [19]. Other studies reported consumer-related barriers (such as issues around privacy and choice [49,55,69]), differences between types of policymaker (such as civil servants vs. managers) [29,70] and public opinion. External events were reported as a facilitator in one study. The role of local context, contingency, and serendipity in influencing policy processes and outcomes overall emerged as a theme throughout the results.

### Comparing with Innvaer (2002): focus of new evidence in the area

There are differences between the reviews (see Table 3), in part reflecting the broader inclusion criteria for this update. However, it is clear that interest in studying the use of evidence has spread beyond the health sector, with more attention from other public policy domains. In addition, there is an increase in publications from low-and middle income countries, where the contexts, barriers and pressures on policymakers in these countries are likely to be very different from those in high-income countries. However, the main research methods used by included studies, and the results generated by those methods, are similar. Despite this increase in research attention, there is still a remarkable dearth of reliable empirical evidence about the actual processes and impacts of research and other evidence use in policy.

**Table 3:** Comparing the two reviews

	**Innvaer [**[10]**]**	**Current systematic review**
Number of studies	24	145
Study designs	Mainly small survey and interview-based studies of policymakers’ perceptions	Mainly small survey and interview-based studies of policymakers’ perceptions with a minority of in-depth case studies
Policy domains	All health	Mainly health, but with studies from a wide range of policy contexts
Countries	Mainly OECD	At least 1/3 from LMIC
Main facilitator	Personal contact between researchers and PMs	Available, clear and relevant research evidence
	Timeliness and relevance of research, with clear recommendations & high quality	Relationships, collaboration & contact between researchers and PMs
	Research confirming current policy	Timing, practical managerial support and
Main barriers	Absence of personal contact between researchers and policymakers	Lack of clear or relevant research evidence, costs
	Lack of timeliness or relevance	Lack of timeliness or opportunity
	Mutual mistrust between scientists and policymakers	Lack of PM research skills or awareness
	Power and budget struggles	

## Discussion

This systematic review aimed to identify and describe research about the barriers and facilitators of the use of evidence for policy, expanding on and updating Innvaer [10]. It found that organisational factors, including availability and access to research were considered to be important influences on whether evidence was used in policy, and the quality of the relationship and collaboration between researchers and policymakers to be the single most mentioned facilitator.

The findings of the updated systematic review presented here were consistent with the original review. We can have a high degree of confidence that it is possible to identify factors likely to influence research uptake, as the expanded field of research synthesised here demonstrates. However, it is less clear what we can learn from this research. For example, there was a high degree of consistency in the findings, even though studies from very different contexts were included. It seems plausible that developing countries would have different barriers from wealthy countries; or that criminal justice would have systematically different pressures from health policy. The similarities reported in these studies may be accounted for by the similarity in approach and methods used. Indeed, the impact and contributions of research to policy (and vice versa) are still unclear, with few studies exploring how, when and why different facilitators and barriers come into play during the policymaking process, or developing an understanding of how research impact on policy and populations might be evaluated. However, there are undoubtedly wider questions about how impact may be defined and measured which are, as yet, unanswered. While perceptions and attitudes are of course important to illuminating the policy process, but there are likely to be other ways - for example documentary, historical, ethnographic or network analyses - in which the role of evidence could be, unpicked [71].

Over a third of the included studies mentioned use of informal evidence such as local data or tacit knowledge. Researchers are starting to recognize that research evidence is just one source of information for policymakers [72]. Identifying these sources and types of information are a crucial step in describing and ultimately influencing the policy process. However, most studies do not define what they mean by evidence’, hampering attempts to understand the process. Interventions addressing barriers specifically are unlikely to influence policy without a detailed understanding of all these factors.

Studies in this area continue to be mainly written by and for researchers, with a lack of attention given to the policy process or policymakers’ priorities. Most studies asked researchers about their perspectives. Where mixed populations were included, the researchers often outnumbered the other participants. Involving policymakers in designing and writing a study which looks at these issues in conjunction with barriers and facilitators may be fruitful. Until then, it is hard to defend academics from the charge of misunderstanding policy priorities or processes – a charge first made explicit over 20 years ago [73].

### Strengths and weaknesses of the study

The review is exhaustive, and we followed a pre-published protocol and rigorous review methods, including the advice of an advisory group (details available from the corresponding author) (see Additional file 3 for a PRISMA checklist report). However, this paper has only counted the frequencies with which factors are mentioned without any weighting. Without more research, it is difficult to say what impact different factors might have.

Most studies still employ relatively superficial methods such as surveys or short interviews. These were all based on self-reports, however, so given the contentious nature of the topic combined with understandable fear of audit/performance monitoring these results may not be reliable. However, there is some evidence that researchers are employing impact assessment, intervention, or observational studies as well to explore how evidence and policy are related. We were unable to double-screen and double-code all studies due to lack of resources. However, all studies were data-extracted at least twice (once at the beginning, and again with the finalized list of factors which was developed iteratively) so we have confidence in the consistency of approach. No methodological assessment of included studies was undertaken, as this was primarily a descriptive exercise. In addition, the heterogeneity of study designs and the difficulty of comparing quality across these domains limited the usefulness of such an exercise. Quality appraisal would be a valuable step in any in-depth review of a subset of these studies.

### Strengths and weaknesses in relation to other studies

Recent systematic reviews in the area have focused on the use of research evidence, [74] or on the impact of research evidence on policy [5]. Orton [75], included in this review, does not include any evaluations of evidence use, ethnographies, or case studies, relying only on self-report questionnaires and interviews to provide the results. Without empirical data exploring access to information and perceived impact [74], and without investigating the policy process, or testing current theories about knowledge utilization, it is hard to draw useful conclusions. Few studies have systematically appraised the use of evidence in this wider sense.

The reviews all found similar findings with regard to barriers and facilitators of the use of evidence. There still appears to be a need for high-quality, simple, clear and relevant research summaries, to be delivered by known and trusted researchers.

### Possible change in future research practice and policymaking

Most studies in this review are descriptive. Because most studies do not go into the content of the facilitators and barriers they identified, we know little about when, why and how the identified barriers and facilitators come into play in the use of evidence in policymaking. Based on this review, future research can use Table 2 to identify themes and factors relevant for their field of research, be it organisations, collaboration, research, researchers, policymakers or policy. Identifying the content and relative importance of these factors and new undiscovered factors in different contexts, at different levels, or in different countries, may contribute to our understanding of evidence use in policy.

One future objective for researchers can be drawn from the results found in Table 1, namely that four of the five top barriers to the use of evidence is a lack of relevance and importance. If research becomes available, the possibility of increased use improves. If policymakers’ research skills improve, calculations of costs will become more accurate. The natural question is to explore why policymakers do not prioritize overcoming barriers relating to themselves. The barrier called lack of clarity, relevance and reliability of research calls for change in researchers’ objectives and methods, but we need to know what policymakers define as clear, relevant and reliable research, and why and when policymakers will use such research. Of special relevance to this question, is the research on knowledge translation done in the past five to ten years, which formed a new strand of research. This body of work draws on the theory that interpersonal relations are important for knowledge exchange, through employing knowledge brokers or similar. There has also been a growth in resources aimed at helping decision-makers to navigate research evidence, such as Cochrane-produced evidence summaries. These are not only aimed at practitioners within the health field, and the knowledge translation field will hopefully soon make efforts at addressing the broader issues around evidence use in policy more widely to identify underlying mechanisms behind knowledge use.

## Conclusion

This review looked for all barriers and facilitators of the used of evidence in policy. Most studies collected research and policy actors’ *perceptions* about factors affecting the use of research evidence, with a large minority surveying only researchers. Understanding how to alleviate these barriers is hampered by a lack of clarity about how evidence’ is defined by studies, with fewer than half specifying what kinds of information were discussed. Most studies however focused on uptake of research evidence, as opposed to evidence more widely. Research into how to alleviate organisational and resource barriers effectively would be welcomed. Additionally, all such research should be based on an understanding that a broader interpretation of “evidence” than “research-based” evidence is also essential.

Stakeholders perceive relationships to be essential elements of the policy process. However, few studies use dedicated relational methods such as network analysis to study policy communities or the policy process, with a few exceptions [75,76].

Several new strands of research offer encouragement to researchers in the area. Firstly, learning from political sciences and management studies is filtering into the EBP debates, as can be seen from the attention paid to leadership and organisational factors. Research into policy entrepreneurship and knowledge brokerage also formed a significant subset of studies. However, there remains a need for empirical evidence to be generated about the policy process. The barriers and facilitators generated above refer specifically to the use of evidence; however, it is equally possible that similar factors affect the policy process in general (for example, constraints on resources, personnel and costs are likely to affect all policy decisions). Identification and exploration of all factors influencing policy, not just those relating to evidence, should be of interest to researchers; however, this is outside the scope of this review.

Finally, little empirical evidence about the processes or impact of the use of evidence by policy is presented by these studies. Despite the increased amount of research on interventions to increase research use in policy, this is not linked with research about the impact of policy on populations, or of evidence use on population outcomes. Much of the literature is concerned with policymaking; but policymakers’ time is spend on implementation. To justify the continuing rhetoric about the importance of research use, and the ever-increasing amount of research into the area, it is surely essential that we practise what we preach and generate evidence about the process and effectiveness of research use in policy.

### “What this paper adds” box

#### ***Section 1: What is already known on this subject***

Little is known about the role of research in policymaking. A previous systematic review (Innvaer [10]) identified the main barriers and facilitators of the use of evidence. Although subsequent reviews have been conducted, they have focused on specific types of evidence, such as economic analyses (Williams) or systematic reviews (Best), or on first-world countries (Orton). Given the explosion of research in the area, an update of the original review was carried out.

#### ***Section 2: What this study add***

The most often mentioned facilitators of the use of evidence are still reported to be relationships, contact and collaboration, availability and access to research, and relevant, reliable and clear research findings. A lack of relevant, reliable and clear research findings, and poor availability and access to research, are the most often mentioned barriers to policymakers’ use of research.

Research into EBP has spread across a wide range of policy areas and countries, including those from low and middle-income countries. New strands of research focus on knowledge translation, knowledge brokerage, and other interventions to increase uptake of evidence. Little research exists about the process, impact or effectiveness of how, when and why research is used during the policy process.

This study did not require ethics approval.

Data sharing: Full dataset and search strategies are available from Kathryn Oliver at Kathryn.oliver@manchester.ac.uk. Consent was not obtained as this study had no participants.

## Competing interests

All authors have completed the Unified Competing Interest form at http://www.icmje.org/coi_disclosure.pdf (available on request from the corresponding author) and declare that none of the authors (KO, SI, TL, JW or JT) have no non-financial interests that may be relevant to the submitted work. All authors declare they have no competing interests.

## Authors’ contribution

KO designed the study, carried out the searches, screened and data-extracted studies, and prepared the manuscript. She is the guarantor. SI provided the search strings, data-extracted studies, helped to analyse the data and helped prepare the manuscript. TL data-extracted studies, helped to analyse the data and prepared the manuscript. JW screened studies, helped design the scope and helped prepare the manuscript. JT helped to analyse the data and helped prepare the manuscript. All authors read and approved the final manuscript.

## Pre-publication history

The pre-publication history for this paper can be accessed here:

http://www.biomedcentral.com/1472-6963/14/2/prepub

## Supplementary Material

Additional file 1 Sample search strategy. Click here for file

Additional file 2 Characteristics of included studies. Click here for file

Additional file 3 Research checklist. Click here for file

## References

[B1] Macintyre S Chalmers I Horton R Smith R Using evidence to inform health policy: case study BMJ 2001 14 222 225 10.1136/bmj.322.7280.222 11159625 PMC1119477

[B2] Nutbeam D Getting evidence into policy and practice to address health inequalities Health Promot Int 2004 14 137 140 15128705 10.1093/heapro/dah201

[B3] Oakley A Hammersley M Evidence-informed policy and practive: challenges for social science Educational Research and Evidence-Based Practive 2007 London: SAGE 91 105

[B4] Pawson R Evidence-Based Policy: A Realist Perspective 2006 London: Sage Publications Ltd

[B5] Boaz A Baeze J Fraser A Effective implementation of research into practice: an overview of systematic reviews of the health literature BMC Res Notes 2011 14 212 21696585 10.1186/1756-0500-4-212 PMC3148986

[B6] Murthy L Shepperd S Clarke M Garner S Lavis J Perrier L Interventions to improve the use of systematic reviews in decision-making by health system managers, policymakers and clinicians Cochrane Database Syst Rev 2012 14 CD009401 22972142 10.1002/14651858.CD009401.pub2 PMC11627148

[B7] Perrier L Mrklas K Lavis J Straus S Interventions encouraging the use of systematic reviews by health policymakers and managers: a systematic review Implementation Sci 2011 14 43 10.1186/1748-5908-6-43 PMC3104485 21524292

[B8] Dobrow MJ Goel V Lemieux-Charles L Black NA The impact of context on evidence utilization: a framework for expert groups developing health policy recommendations Soc Sci Med 2006 14 1811 1824 10.1016/j.socscimed.2006.04.020 16764980

[B9] Morrato E Elias M Gericke C Using population-based routine data for evidence-based health policy decisions: lessons from three examples of setting and evaluating national health policy in Australia, the UK and the USA J Public Health 2008 14 4 463 471 10.1093/pubmed/fdm065 17942850

[B10] Innvaer S Vist G Trommald M Oxman A Health policymakers’ perceptions of their use of evidence: a systematic review J Health Serv Res Policy 2002 14 239 244 10.1258/135581902320432778 12425783

[B11] Lavis J Davies H Oxman A Denis JL Golden-Biddle K Ferlie E Towards systematic reviews that inform health care management and policymaking J Health Serv Res Policy 2005 14 35 48 10.1258/1355819054308549 16053582

[B12] Orton L Lloyd-Williams F Taylor-Robinson D O’Flaherty M Capewell S The use of research evidence in public health decision making processes: systematic review PLoS One 2011 14 e21704 10.1371/journal.pone.0021704 21818262 PMC3144216

[B13] Thomas J Brunton J EPPI-Reviewer 3.0: Analysis and Management of Data for Research Synthesis. EPPI-Centre Software. [3.0] 2006 London: Social Science Research Unit, Institute of Education Ref Type: Computer Program

[B14] Gagliardi AR Fraser N Wright FC Lemieux-Charles L Davis D Fostering knowledge exchange between researchers and decision-makers: exploring the effectiveness of a mixed-methods approach Health Policy 2008 14 53 63 10.1016/j.healthpol.2007.09.002 17935826

[B15] Department of Health Saving Lives: Our Healthier Nation 1999 London: HMSO Ref Type: Report

[B16] Learmonth AM Utilizing research in practice and generating evidence from practice Health Educ Res 2000 14 743 756 10.1093/her/15.6.743 11142081

[B17] Hinchcliff R Ivers R Poulos R Senserrick T Utilization of research in policymaking for graduated driver licensing Am J Public Health 2010 14 2052 2058 10.2105/AJPH.2009.184713 20864713 PMC2951929

[B18] Hinchcliff R Poulos R Ivers RQ Senserrick T Understanding novice driver policy agenda setting Public Health 2011 14 217 221 10.1016/j.puhe.2011.01.001 21440272

[B19] Anaraki S Plugge E Delivering primary care in prison: the need to improve health information Inform Prim Care 2003 14 191 194 14980057 10.14236/jhi.v11i4.566

[B20] Bédard P Ouimet M Cognizance and consultation of randomized controlled trials among ministerial policy analysts Rev Policy Res 2012 14 625 644 10.1111/j.1541-1338.2012.00581.x

[B21] Henderson CE Young DW Farrell J Taxman FS Associations among state and local organizational contexts: Use of evidence-based practices in the criminal justice system Drug Alcohol Depend 2009 14 Suppl 1 S23 S32 19174321 10.1016/j.drugalcdep.2008.12.006 PMC4934022

[B22] Jennings ET Hall JL Evidence-based practice and the use of information in state agency decision making J Public Adm Res Theory 2012 14 245 266 10.1093/jopart/mur040

[B23] Stevens A Telling policy stories: an ethnographic study of the use of evidence in policymaking in the UK J Soc Policy 2011 14 237 255 10.1017/S0047279410000723

[B24] Aaserud M Lewin S Innvaer S Paulsen E Dahlgren A Trommald M Translating research into policy and practice in developing countries: a case study of magnesium sulphate for pre-eclampsia BMC Health Serv Res 2005 14 68 10.1186/1472-6963-5-68 16262902 PMC1298297

[B25] Albert MA Fretheim A Maiga D Factors influencing the utilization of research findings by health policymakers in a developing country: the selection of Mali’s essential medicines Health Res Policy Syst 2007 14 2 10.1186/1478-4505-5-2 17338810 PMC1820594

[B26] Bickford J Kothari A Research and knowledge in Ontario tobacco control networks Canadian J Public Health Rev Can Sante Publique 2008 14 297 300 10.1007/BF03403759 PMC6975625 18767275

[B27] Burris H Parkhurst J Du-Sarkodie Y Mayaud P Getting research into policy - herpes simplex virus type-2 (HSV-2) treatment and HIV infection: international guidelines formulation and the case of Ghana Health Res Policy Syst 2011 14 Suppl 1 S5 10.1186/1478-4505-9-S1-S5 21679386 PMC3121136

[B28] Currie L Clancy L The road to smoke-free legislation in Ireland. [References] Addiction 2011 14 15 24 10.1111/j.1360-0443.2010.03157.x 20955215

[B29] Frey K Widmer T Revising swiss policies: the influence of efficiency analyses Am J Eval 2011 14 494 517 10.1177/1098214011401902

[B30] Greyson DL Cunningham C Morgan S Information behaviour of Canadian pharmaceutical policymakers Health Info Libr J 2012 14 16 27 10.1111/j.1471-1842.2011.00969.x 22335286

[B31] Hutchinson E Parkhurst J Phiri S Gibb DM Chishinga N Droti B National policy development for cotrimoxazole prophylaxis in Malawi, Uganda and Zambia: the relationship between context, evidence and links Health Res Policy Syst 2011 14 Suppl 1 S6 10.1186/1478-4505-9-S1-S6 21679387 PMC3121137

[B32] Innvaer S The use of evidence in public governmental reports on health policy: an analysis of 17 Norwegian official reports (NOU) BMC Health Serv Res 2009 14 177 10.1186/1472-6963-9-177 19785760 PMC2761392

[B33] Kurko T Silvast A Wahlroos H Pietila K Airaksinen M Is pharmaceutical policy evidence-informed? A case of the deregulation process of nicotine replacement therapy products in Finland Health Policy 2012 14 246 255 10.1016/j.healthpol.2012.02.013 22417863

[B34] Rieckmann TR Kovas AE Cassidy EF McCarty D Employing policy and purchasing levers to increase the use of evidence-based practices in community-based substance abuse treatment settings: Reports from single state authorities Eval Program Plann 2011 14 366 374 10.1016/j.evalprogplan.2011.02.003 21371753 PMC3670771

[B35] Ritter A How do drug policymakers access research evidence? Int J Drug Policy 2009 14 70 75 10.1016/j.drugpo.2007.11.017 18226519

[B36] Rocchi A Menon D Verma S Miller E The role of economic evidence in Canadian oncology reimbursement decision-making: to lambda and beyond Value Health 2008 14 771 783 10.1111/j.1524-4733.2007.00298.x 18179658

[B37] Wang A Baerwaldt T Kuan R Nordyke R Halbert R Payer perspectives on evidence for formulary decision making in the United States Value Health 2011 14 A350

[B38] Carneiro M Silva-Rosa T The use of Scientific Knowledge in the Decision Making Process of Environmental Public Policies in Brazil 2011 Science Communication: Journal of 10

[B39] Comptroller and Auditor General of the National Audit Office Getting the Evidence: Using Research in Policymaking 2003 London: Stationery Office

[B40] Deelstra Y Nooteboom SG Kohlmann HR Berg J Innanen S Using knowledge for decision-making purposes in the context of large projects in The Netherlands Environ Impact Assess Rev 2003 14 517 541 10.1016/S0195-9255(03)00070-2

[B41] Ortega-Argueta A Baxter G Hockings M Compliance of Australian threatened species recovery plans with legislative requirements J Environ Manage 2011 14 2054 2060 10.1016/j.jenvman.2011.03.032 21507558

[B42] Weitkamp G Van den Berg AE Bregt AK Van Lammeren RJA Evaluation by policymakers of a procedure to describe perceived landscape openness J Environ Manage 2012 14 17 28 10.1016/j.jenvman.2011.09.022 22115507

[B43] Lomas J Brown A Research and advice giving: a functional view of evidence-informed policy advice in a canadian ministry of health Milbank Q 2009 14 903 926 10.1111/j.1468-0009.2009.00583.x 20021590 PMC2888020

[B44] Bryce J Victora C Habicht J Vaghan J Black R The multi-country evaluation of the integrated management of childhood illness strategy: lessons for the evaluation of public health interventions Am J Public Health 2004 14 94 105 10.2105/ajph.94.3.406 PMC1448266 14998804

[B45] Hivon ML Use of health technology assessment in decision making: coresponsibility of users and producers? Int J Technol Assess Health Care 2005 14 268 275 15921069

[B46] Flitcroft K Gillespie J Salkeld G Carter S Trevena L Getting evidence into policy: the need for deliberative strategies? Soc Sci Med 2011 14 1039 1046 10.1016/j.socscimed.2011.01.034 21419539

[B47] Galani C Self-reported healthcare decision-makers’ attitudes towards economic evaluations of medical technologies Curr Med Res Opin 2008 14 3049 3058 10.1185/03007990802442695 18826747

[B48] Lencucha R Kothari AR Hamel N Extending collaborations for knowledge translation: lessons from the community-based participatory research literature Evid Policy 2010 14 75

[B49] Brambila C Ottolenghi E Marin C Bertrand J Getting results used: evidence from reproductive health programmatic research in Guatemala Health Policy Plan 2007 14 234 245 10.1093/heapol/czm013 17475627

[B50] Hamel N Schrecker T Unpacking capacity to utilize research: a tale of the burkina faso public health association Soc Sci Med 2011 14 31 38 10.1016/j.socscimed.2010.09.051 21074923

[B51] Bunn F Strategies to promote the impact of systematic reviews on healthcare policy: a systematic review of the literature Evid Policy 2011 14 428

[B52] Campbell D Donald B Moore G Frew D Evidence check: knowledge brokering to commission research reviews for policyAN - 857120904; 4175308 Evid Policy 2011 14 97 107 10.1332/174426411X553034

[B53] Dobbins M Robeson P Ciliska D Hanna S Cameron R O’ Mara L A description of a knowledge broker role implemented as part of a randomized controlled trial evaluating three knowledge translation strategies Implementation Science 2009 14 1 16 10.1186/1748-5908-4-23 PMC2680804 19397820

[B54] El-Jardali F Lavis JN Ataya N Jamal D Use of health systems and policy research evidence in the health policymaking in eastern Mediterranean countries: views and practices of researchers Implementation Science 2012 14 2 10.1186/1748-5908-7-2 22236561 PMC3286421

[B55] Jack SM Knowledge transfer and exchange processes for environmental health issues in Canadian Aboriginal communities Int J Environ Res Public Health 2010 14 651 674 10.3390/ijerph7020651 20616996 PMC2872293

[B56] Jonsson K Health systems research in Lao PDR: capacity development for getting research into policy and practice Health Res Policy Syst 2007 14 10.1186/1478-4505-5-11 PMC2098768 17939854

[B57] Ward V Smith S House A Hamer S Exploring knowledge exchange: a useful framework for practice and policy Soc Sci Med 2012 14 297 304 10.1016/j.socscimed.2011.09.021 22014420

[B58] Williams I McIver S Moore D Bryan S The use of economic evaluations in NHS decision-making: a review and empirical investigation Health Technol Assess 2008 14 iii-175 10.3310/hta12070 18373906

[B59] Friese B Bogenschneider K The voice of experience: How social scientists communicate family research to policymakers Family Relations 2009 14 229 243 10.1111/j.1741-3729.2008.00549.x 20407597 PMC2856091

[B60] Petticrew M Whitehead M Macintyre SJ Graham H Egan M Evidence for public health policy on inequalities: 1: the reality according to policymakers J Epidemiol Community Health 2004 14 811 816 10.1136/jech.2003.015289 15365104 PMC1763325

[B61] Smith K Joyce K Capturing complex realities: understanding efforts to achieve evidence-based policy and practice in public health Evidence and Policy 2012 14 78

[B62] Bunn F Kendall S Does nursing research impact on policy? A case study of health visiting research and UK health policy. [References] J Res Nurs 2011 14 169 191 10.1177/1744987110392627

[B63] Hyder AA Corluka A Winch PJ El-Shinnawy A Ghassany H Malekafzali H National policymakers speak out: are researchers giving them what they need? Health Policy Plan 2011 14 73 82 10.1093/heapol/czq020 20547652 PMC4031573

[B64] Hird JA Policy analysis for what? The effectiveness of nonpartisan policy research organizations Policy Stud J 2005 14 83 105 10.1111/j.1541-0072.2005.00093.x

[B65] Olson B Armstrong EP Grizzle AJ Nichter MA Industry’s perception of presenting pharmacoeconomic models to managed care organizations J Manag Care Pharm 2003 14 159 167 14613345 10.18553/jmcp.2003.9.2.159 PMC10437175

[B66] Brownson RC Dodson EA Stamatakis KA Casey CM Elliott MB Luke DA Communicating evidence-based information on cancer prevention to state-level policymakers J Natl Cancer Inst 2011 14 306 316 10.1093/jnci/djq529 21212381 PMC3039727

[B67] Larsen M Gulis G Pedersen KM Use of evidence in local public health work in Denmark Int J Public Health 2012 14 477 483 10.1007/s00038-011-0324-y 22116391

[B68] Dobbins M Cockerill R Barnsley J Ciliska D Factors of the innovation, organization, environment, and individual that predict the influence five systematic reviews had on public health decisions Int J Technol Assess Health Care 2001 14 467 478 11758291

[B69] Jewell CJ Bero LA “Developing good taste in evidence”: facilitators of and hindrances to evidence-informed health policymaking in state government Milbank Q 2008 14 177 208 10.1111/j.1468-0009.2008.00519.x 18522611 PMC2690362

[B70] McDavid JC Huse I Legislator uses of public performance reports: Findings from a five-year study Am J Eval 2012 14 7 25 10.1177/1098214011405311

[B71] Wieshaar H Collin J Smith K Gruning T Mandal S Gilmore A Global health governance and the commercial sector: a documentary analysis of tobacco company strategies to influence the WHO framework convention on tobacco control PLoS Med 2012 14 e1001249 10.1371/journal.pmed.1001249 22745607 PMC3383743

[B72] Haynes AS Gillespie JA Derrick GE Hall WD Redman S Chapman S Galvanizers, guides, champions, and shields: the many ways that policymakers use public health researchers Milbank Q 2011 14 564 598 10.1111/j.1468-0009.2011.00643.x 22188348 PMC3250634

[B73] Weiss C The many meanings of research utilisation Public Adm Rev 1979 14 426 431 10.2307/3109916

[B74] Orton L The Use of research evidence in public health decision making processes: systematic review PLoS One 2011 14 e21704 10.1371/journal.pone.0021704 21818262 PMC3144216

[B75] Lewis JM Being around and knowing the players: networks of influence in health policy Soc Sci Med 2006 14 2125 2136 10.1016/j.socscimed.2005.10.004 16289737

[B76] Oliver K de Vocht F Money A Everett MG Who runs public health? A mixed-methods study combining network and qualitative analyses J Public Health 2013 14 453 459 10.1093/pubmed/fdt039 23564840

