# Supplementary material for: A systematic review of barriers to and facilitators of the use of evidence by policymakers
Source: BMC Health Serv Res. 2014 Jan 3;14:2. doi: 10.1186/1472-6963-14-2 (PMC3909454; doi:10.1186/1472-6963-14-2)
Supplement: Additional file 1 — Sample search strategy. [file 1472-6963-14-2-S1.docx]

Sample search strategy

1. health care policy/

2. decision making/

3. medical decision making/

4. POLICY/

5. MANAGEMENT/

6. health polic$.tw.

7. decision$.tw.

8. (policy mak$ or policymak$).tw.

9. public polic$.tw.

10. health plan$.tw.

11. evidence$.tw.

12. (evidence$ adj6 health polic$).tw.

13. (evidence$ adj2 decision$).tw.

14. (evidence$ adj6 (policy mak$ or policymak$)).tw.

15. (evidence$ adj6 public polic$).tw.

16. (evidence$ adj6 health plan$).tw.

17. (research$ adj6 health polic$).tw.

18. (research$ adj2 decision$).tw.

19. (research$ adj6 (policy mak$ or policymak$)).tw.

20. (research$ adj6 public polic$).tw.

21. (research$ adj6 health plan$).tw.

22. ((science$ or scient$) adj2 health polic$).tw.

23. ((scienc$ or scient$) adj2 (policy mak$ or policymak$)).tw.

24. ((scienc$ or scient$) adj2 public polic$).tw.

25. ((scienc$ or scient$) adj2 health plan$).tw.

26. (information$ adj2 (policy mak$ or policymak$)).tw.

27. (information$ adj2 health polic$).tw.

28. (information$ adj2 health plan$).tw.

29. (information$ adj2 public polic$).tw.

30. (data$ adj2 health plan$).tw.

31. (data$ adj2 (policy mak$ or policymak$)).tw.

32. (data$ adj2 health polic$).tw.

33. (data$ adj2 public polic$).tw.

34. (evidence$ adj6 (barrier$ or facilitator$ or uptake$ or utilis$ or utiliz$ or diffus$ or disseminat$)).tw.

35. (research adj6 (barrier$ or facilitator$ or uptake$ or utilis$ or utiliz$ or diffus$ or disseminat$)).tw.

36. (information adj6 (barrier$ or facilitator$ or uptake$ or utilis$ or utiliz$ or diffus$ or disseminat$)).tw.

37. (data adj2 (barrier$ or facilitator$ or uptake$ or utilis$ or utiliz$ or diffus$ or disseminat$)).tw.

38. 12 or 13 or 14 or 15 or 16 or 17 or 18 or 19 or 20 or 21 or 22 or 23 or 24 or 25 or 26 or 27 or 28 or 29 or 30 or 31 or 32 or 33

39. 34 or 35 or 36 or 37

40. 1 or 2 or 3 or 4 or 5 or 6 or 7 or 8 or 9 or 10

41. 38 or 39

42. 40 and 41

43. 38 or 40

44. 39 and 43

45. limit 44 to (human and english language and yr="2000 - 2010")
