# Supplementary material for: A systematic review of barriers to and facilitators of the use of evidence by policymakers
Source: BMC Health Serv Res. 2014 Jan 3;14:2. doi: 10.1186/1472-6963-14-2 (PMC3909454; doi:10.1186/1472-6963-14-2)
Supplement: Additional file 2 — Characteristics of included studies. [file 1472-6963-14-2-S2.docx]

| **Item** | **Methods** | **Population** | **Results: Barriers** | **Results: Facilitators** | **Results: Other** |
| --- | --- | --- | --- | --- | --- |
| Aaserud (2005) (1)  **Study design** Case study | **Time frame** Cross-sectional  **Data collection** Survey  Group interviews  Observation - written notes  **Data analysis** Thematic analysis  Descriptive statistics | **Sample population** Researchers  Physicians  Surgeons  Midwives  Health care managers  Information/surveillance staff  **Setting** Other  **Discipline** drugs policy | **Barriers to use of evidence** Contact with policy makers  Costs  Staff or personnel resources  Managerial will  Political support (will)  Lack of material resources  Lack of legal support  Consumer-related barrier  Policy-maker research skills  Policy-maker research awareness  Practitioner research awareness  Guidelines or policy statement  Other | **Facilitators to use of evidence** Contact with policy makers  Relationship with policy-makers  Political support (will)  Legal support  Practitioner research skills  opportunity/time to influence  professional bodies  Improved dissemination  Guidelines or policy statement  importance of policy | **Types of evidence used** Not stated  **Types of result** suggestions/proposed factors  Perceptions  Intentions  Experiences |
| Albert (2007) (2)  **Study design** Case study  Qualitative study | **Time frame** Cross-sectional  **Data collection** Semi-structured interviews  Group interviews  Documentary analysis  **Data analysis** phenomenological approach | **Sample population** GPs  Allied health professionals  Policy advisor  Health care managers  Information/surveillance staff  **Setting** Policy environment  **Discipline** drugs policy | **Barriers to use of evidence** contact with researchers/info staff  relationship with researchers/info staff  Timing/opportunity  Staff or personnel resources  Availability of research/access to research or info  Ambiguity/relevance/reliability of findings  Lack of legal support  Guidelines or policy statement  Other  importance of research findings | **Facilitators to use of evidence** contact with researchers/info staff  relationship with researchers/info staff  Collaboration  Managerial support (practical)  Availability of research/access to research or info  Clarity/relevance/reliability of research findings  Format of research findings  Policy-maker research skills  opportunity/time to influence  professional bodies  Improved dissemination | **Types of evidence used** Not stated  **Types of result** suggestions/proposed factors  Experiences |
| Anaraki (2003) (3)  **Study design** Qualitative study | **Time frame** Cross-sectional  **Data collection** Survey  Semi-structured interviews  Group interviews  **Data analysis** Not stated | **Sample population** GPs  Nurses  Health care managers  Other  **Setting** Other  **Discipline** Primary care  criminal justice | **Barriers to use of evidence** Other | **Facilitators to use of evidence** Costs  Managerial support (practical)  Availability of research/access to research or info | **Types of evidence used** Other information - not formal research  **Types of result** suggestions/proposed factors  Perceptions |
| Aoki-Suzuki (2012) (4)  **Study design** Quantitative study  Qualitative study | **Time frame** Cross-sectional  **Data collection** Survey  Semi-structured interviews  Focus Group  Documentary analysis  **Data analysis** Descriptive statistics | **Sample population** Policy-makers  **Setting** Policy environment  **Discipline** Other | **Barriers to use of evidence** Lack of material resources  Availability of research/access to research or info  Policy-maker research skills  Policy-maker research awareness  Other |  | **Types of evidence used** Other information - not formal research  **Types of result** Perceptions |
| Armstrong (2006) (5)  **Study design** Qualitative study | **Time frame** Cross-sectional  **Data collection** not stated  **Data analysis** Not stated | **Sample population** Other  **Setting** Not stated  **Discipline** Health policy  public health | **Barriers to use of evidence** Availability of research/access to research or info  Format of research findings  Practitioner research skills  Other |  | **Types of result** Perceptions |
| Babu (2000) (6)  **Study design** Case study | **Time frame** Cross-sectional  **Data collection** OTher  **Data analysis** Not stated | **Sample population** Researchers **Sample population** Policy-makers **Sample population** Other  **Setting** Not stated  **Discipline** Other | **Barriers to use of evidence** Availability of research/access to research or info  Format of research findings | **Facilitators to use of evidence** Collaboration | **Types of evidence used** Primary research  **Types of result** Experiences |
| Baernholdt (2007) (7)  **Study design** Quantitative study | **Time frame** Cross-sectional  **Data collection** Survey  **Data analysis** Descriptive statistics | **Sample population** Nurses  Policy-makers  Other  **Setting** Policy environment  **Discipline** Secondary & tertiary care | **Barriers to use of evidence** Collaboration  Timing/opportunity  Staff or personnel resources  Political support (will)  Availability of research/access to research or info  Ambiguity/relevance/reliability of findings  Policy-maker research awareness  Practitioner research skills  Guidelines or policy statement |  | **Types of evidence used** Not stated  **Types of result** Perceptions  Experiences |
| Baghbanian (2012) (8)  **Study design** Quantitative study  Qualitative study | **Time frame** Cross-sectional  **Data collection** Semi-structured interviews  **Data analysis** Not stated | **Sample population** Policy-makers  Health care managers  **Setting** Health care  **Discipline** Primary care  Health policy  Secondary & tertiary care |  | **Facilitators to use of evidence** relationship with researchers/info staff Availability of research/access to research or info | **Types of evidence used** Not stated  **Types of result** Perceptions |
| Barratt (2003) (9)  **Study design** Qualitative study | **Time frame** Longitudinal  **Data collection** Survey  Semi-structured interviews OTher  **Data analysis** Thematic analysis  Descriptive statistics | **Sample population** Policy-makers  Local authority staff  **Setting** Other  **Discipline** social care / social work | **Barriers to use of evidence** Relationship with policy-makers  Collaboration  Timing/opportunity  Staff or personnel resources  Managerial wil  Managerial support (practical)  Availability of research/access to research or info  Ambiguity/relevance/reliability of findings  Practitioner research skills  Guidelines or policy statement  Other | **Facilitators to use of evidence** Collaboration  Staff or personnel resources  Clarity/relevance/reliability of research findings  Format of research findings  professional bodies | **Types of evidence used** Systematic reviews  Guidelines  Primary research  **Types of result** suggestions/proposed factors  Perceptions  Intentions  Experiences |
| Bédard (2012) (10)  **Study design** Quantitative study | **Time frame** Cross-sectional  **Data collection** Survey  **Data analysis** Descriptive statistics  Regression analysis | **Sample population** Policy advisor  **Setting** Policy environment  **Discipline** Health policy  health promotion  Education  criminal justice  Other  Conservation & environmental management  social care / social work  transport  public health | **Barriers to use of evidence** Policy-maker research skills  Policy-maker research awareness | **Facilitators to use of evidence** contact with researchers/info staff | **Types of evidence used** Primary research  **Types of result** Perceptions |
| Behague (2009) (11)  **Study design** Case study | **Time frame** Cross-sectional  **Data collection** Semi-structured interviews  **Data analysis** Thematic analysis  Content analysis | **Sample population** Researchers  Physicians  Policy-makers  Health care managers  Other  **Setting** Policy environment  **Discipline** Secondary & tertiary care | **Barriers to use of evidence** relationship with researchers/info staff Availability of research/access to research or info  Ambiguity/relevance/reliability of findings  Other  Professional/international bodies  importance of policy | **Facilitators to use of evidence** Contact with policy makers  Collaboration | **Types of evidence used** Primary research  Other information - not formal research  **Types of result** Perceptions  Experiences |
| Ben-Arieh (2008) (12)  **Study design** Case study | **Time frame** Cross-sectional  **Data collection** Semi-structured interviews  **Data analysis** Descriptive statistics | **Sample population** Policy-makers  Other  **Setting** Not stated  **Discipline** Other | **Barriers to use of evidence** Timing/opportunity | **Facilitators to use of evidence** Collaboration  Timing/opportunity  Availability of research/access to research or info  Clarity/relevance/reliability of research findings  Format of research findings  Improved dissemination | **Types of result** suggestions/proposed factors  Perceptions  Experiences |
| Best (2012) (13)  **Study design** Systematic review | **Time frame** Cross-sectional  **Data collection** OTher  **Data analysis** Realist | **Sample population** Other  **Setting** Not applicable  **Discipline** Primary care  Health policy  Secondary & tertiary care |  | **Facilitators to use of evidence** contact with researchers/info staff  Contact with policy makers  relationship with researchers/info staff | **Types of evidence used** Not stated |
| Bickford (2008) (14)  **Study design** Qualitative study | **Time frame** Cross-sectional  **Data collection** Semi-structured interviews  **Data analysis** Content analysis | **Sample population** Policy-makers **Sample population** Other  **Setting** Policy environment  **Discipline** drugs policy | **Barriers to use of evidence** Timing/opportunity  Ambiguity/relevance/reliability of findings  Other | **Facilitators to use of evidence** relationship with researchers/info staff Continuity of employment  professional bodies  Other | **Types of evidence used** Primary research  **Types of result** Perceptions  Experiences |
| Blackman (2012) (15)  **Study design** Qualitative study | **Time frame** Cross-sectional  **Data collection** Semi-structured interviews  **Data analysis** Other | **Sample population** Local authority staff  Other  **Setting** Health care  Policy environment  **Discipline** Health policy | **Barriers to use of evidence** Other |  | **Types of evidence used** Primary research  Other information - not formal research  **Types of result** Perceptions  Experiences |
| Blume (2010)  (16)  **Study design** other | **Time frame** Longitudinal  **Data collection** Documentary analysis  **Data analysis** Content analysis | **Sample population** Other  **Setting** Policy environment  **Discipline** public health | **Barriers to use of evidence** Ambiguity/relevance/reliability of findings | **Facilitators to use of evidence** Availability of research/access to research or info | **Types of evidence used** Primary research  Other information - not formal research  **Types of result** Documentary proof of research use |
| Boaz (2002) (17)  **Study design** Case study | **Time frame** Cross-sectional  **Data collection** not stated  **Data analysis** Not stated | **Sample population** Researchers  Policy-makers  Other  **Setting** Policy environment  **Discipline** social care / social work | **Barriers to use of evidence** Timing/opportunity  Policy-maker research skills | **Facilitators to use of evidence** contact with researchers/info staff  Contact with policy makers  relationship with researchers/info staff  Relationship with policy-makers  Collaboration  Managerial support (practical)  Other | **Types of evidence used** Primary research  **Types of result** Perceptions |
| Brambila (2007) (18)  **Study design** other | **Time frame** Cross-sectional  **Data collection** Semi-structured interviews  Documentary analysis  OTher  **Data analysis** Not stated | **Sample population** Other  **Setting** Health care  **Discipline** Other | **Barriers to use of evidence** Turnover of staff  Ambiguity/relevance/reliability of findings  Consumer-related barrier  Policy-maker research skills | **Facilitators to use of evidence** Relationship with policy-makers  Collaboration  Timing/opportunity  Continuity of employment  Political support (will)  Political support (practical)  Managerial support (practical)  Format of research findings  Policy-maker research skills  Other  importance of research findings | **Types of evidence used** Primary research  **Types of result** Experiences |
| Brownson (2011) (19)  **Study design** Quantitative study | **Time frame** Cross-sectional  **Data collection** Survey  **Data analysis** Descriptive statistics | **Sample population** Policy advisor  Policy-makers  Legal staff  **Setting** Health care  Policy environment  **Discipline** Health policy  Secondary & tertiary care |  | **Facilitators to use of evidence** Clarity/relevance/reliability of research findings  Other | **Types of evidence used** Guidelines  **Types of result** Perceptions |
| Bryce (2004)  (20)  **Study design** other | **Time frame**  Cross-sectional  **Data collection** not stated  **Data analysis** Not stated | **Sample population** Other  **Setting** Policy environment  **Discipline** Primary care | **Barriers to use of evidence** Collaboration  Turnover of staff  Ambiguity/relevance/reliability of findings  Professional/international bodies  importance of research findings | **Facilitators to use of evidence** Contact with policy makers  Relationship with policy-makers  Timing/opportunity  Availability of research/access to research or info  Clarity/relevance/reliability of research findings  professional bodies  Improved dissemination  Other | **Types of evidence used** Not stated  **Types of result** Perceptions |
| Bunn (2011) (21)  **Study design** Systematic review | **Time frame** Cross-sectional  **Data collection** Other  **Data analysis** Not stated | **Sample population** Other  **Setting** Not applicable  **Discipline** Primary care  Health policy  Secondary & tertiary care | **Barriers to use of evidence** Timing/opportunity  Staff or personnel resources  Availability of research/access to research or info  Ambiguity/relevance/reliability of findings  Format of research findings  Policy-maker research skills  Policy-maker research awareness  Other | **Facilitators to use of evidence** relationship with researchers/info staff  Relationship with policy-makers  Collaboration  Clarity/relevance/reliability of research findings  Format of research findings  Practitioner research skills  Improved dissemination  Other | **Types of evidence used** Systematic reviews  **Types of result** Perceptions  Experiences |
| Bunn (2011) (22)  **Study design** Case study  Qualitative study | **Time frame** Cross-sectional  **Data collection** Semi-structured interviews  Documentary analysis  **Data analysis** Content analysis | **Sample population** Researchers  **Setting** Health care  **Discipline** Primary care  transport | **Barriers to use of evidence** Timing/opportunity  Availability of research/access to research or info  Other | **Facilitators to use of evidence** Timing/opportunity  Availability of research/access to research or info | **Types of evidence used** Guidelines  Primary research  Other information - not formal research  **Types of result** Documentary proof of research use  Perceptions |
| Burris (2011) (23)  **Study design** Case study | **Time frame** Cross-sectional  **Data collection** Semi-structured interviews  **Data analysis** Content analysis | **Sample population** Researchers  Policy advisor  Policy-makers  Other  **Setting** Health care  **Discipline** Health policy  drugs policy | **Barriers to use of evidence** Timing/opportunity  Costs  Lack of material resources  Policy-maker research awareness  Other | **Facilitators to use of evidence** contact with researchers/info staff  Contact with policy makers  relationship with researchers/info staff  Relationship with policy-makers  Collaboration  Timing/opportunity  Costs  Material resources available  Political support (will)  Political support (practical) | **Types of evidence used** Not stated  **Types of result** Perceptions  Experiences |
| Cameron (2011) (24)  **Study design** Case study Qualitative study | T**ime frame** Cross-sectional  **Data collection** Semi-structured interviews  Documentary analysis  **Data analysis** Thematic analysis  Content analysis | **Sample population** Policy advisor  Policy-makers  **Setting** Policy environment  **Discipline** Health policy | **Barriers to use of evidence** Timing/opportunity  Ambiguity/relevance/reliability of findings | **Facilitators to use of evidence** Continuity of employment  Clarity/relevance/reliability of research findings  Improved dissemination | **Types of evidence used** Primary research  Other information - not formal research  **Types of result** Perceptions  Intentions  Experiences |
| Campbell (2006) (25)  **Study design** Qualitative study | **Time frame** Cross-sectional  **Data collection** Survey **Data collection** Semi-structured interviews  **Data analysis** Thematic analysis **Data analysis** Descriptive statistics | **Sample population** Researchers **Sample population** Policy-makers  **Setting** Academic institution **Setting** Policy environment  **Discipline** Health policy | **Barriers to use of evidence** Timing/opportunity  Costs  Lack of material resources  Availability of research/access to research or info  Format of research findings  Policy-maker research skills  Policy-maker research awareness  Other  Professional/international bodies  importance of policy  importance of research findings | **Facilitators to use of evidence** contact with researchers/info staff  Contact with policy makers  Relationship with policy-makers  Collaboration  Timing/opportunity  Availability of research/access to research or info  Clarity/relevance/reliability of research findings  Policy-maker research awareness  Policy-maker research skills  Guidelines or policy statement  Other | **Types of evidence used** Systematic reviews  Other information - not formal research  **Types of result** suggestions/proposed factors  Perceptions  Experiences |
| Campbell (2011) (26)  **Study design** Case study | **Time frame** Cross-sectional  **Data collection** Semi-structured interviews  **Data analysis** Thematic analysis | **Sample population** Researchers  Policy advisor  Policy-makers  **Setting** Policy environment  **Discipline** Primary care  Health policy  Secondary & tertiary care public health |  | **Facilitators to use of evidence** contact with researchers/info staff  relationship with researchers/info staff Clarity/relevance/reliability of research findings Other | **Types of evidence used** Systematic reviews  **Types of result** Perceptions  Experiences |
| Carneiro (2011) (27)  **Study design** Qualitative study | **Time frame** Cross-sectional  **Data collection** Semi-structured interviews  **Data analysis** Thematic analysis | **Sample population** Researchers  Local authority staff  Other  Legal staff  **Setting** Policy environment  **Discipline** Conservation & environmental management | **Barriers to use of evidence** Timing/opportunity  Lack of material resources  Availability of research/access to research or info  Ambiguity/relevance/reliability of findings  Format of research findings  Other | **Facilitators to use of evidence** contact with researchers/info staff | **Types of evidence used** Not stated  **Types of result** Perceptions  Experiences |
| Cerveny (2011) (28)  **Study design** Quantitative study | **Time frame** Cross-sectional  **Data collection** Survey  Semi-structured interviews  **Data analysis** Descriptive statistics | **Sample population** Other  **Setting** Other  **Discipline** Conservation & environmental management |  | **Facilitators to use of evidence** Availability of research/access to research or info  Clarity/relevance/reliability of research findings  Other | **Types of evidence used** Other information - not formal research  **Types of result** Perceptions |
| Chambers (2011) (29)  **Study design** Systematic review | **Time frame** Cross-sectional  **Data collection** OTher  **Data analysis** Descriptive - no analysis | **Sample population** Other  **Setting** Health care  **Discipline** Primary care  Health policy  Secondary & tertiary care |  | **Facilitators to use of evidence** Clarity/relevance/reliability of research findings  Format of research findings | **Types of evidence used** Systematic reviews  **Types of result** Other |
| Chambers (2012) (30)  **Study design** Case study | **Time frame** Cross-sectional  **Data collection** Survey  **Data analysis** Not stated | **Sample population** Health care managers  **Setting** Health care  **Discipline** Primary care  Secondary & tertiary care | **Barriers to use of evidence** Timing/opportunity | **Facilitators to use of evidence** Contact with policy makers  Format of research findings | **Types of evidence used** Systematic reviews  **Types of result** Perceptions |
| Coleman (2001) (31)  **Study design** Quantitative study  Qualitative study | **Time frame** Cross-sectional  **Data collection** Survey  **Data analysis** Descriptive statistics | **Sample population** GPs  Physicians  Surgeons  Policy-makers  Health care managers  Other  **Setting** Health care  **Discipline** Primary care  Health policy  Secondary & tertiary care |  | **Facilitators to use of evidence** Availability of research/access to research or info  Clarity/relevance/reliability of research findings  Format of research findings  Improved dissemination | **Types of evidence used** Systematic reviews  Guidelines  Primary research  Other information - not formal research  **Types of result** Behaviour |
| Colon-Ramos (2007) (32)  **Study design** Case study  Qualitative study | **Time frame** Cross-sectional  **Data collection** Semi-structured interviews  Documentary analysis  **Data analysis** Thematic analysis | **Sample population** Researchers  Policy-makers  Other  **Setting** Not stated  **Discipline** Other  public health | **Barriers to use of evidence** relationship with researchers/info staff  Relationship with policy-makers  Availability of research/access to research or info  Ambiguity/relevance/reliability of findings  Consumer-related barrier  Policy-maker research skills  Policy-maker research awareness  Professional/international bodies  importance of policy  importance of research findings | **Facilitators to use of evidence** Collaboration | **Types of evidence used** Primary research  Other information - not formal research  **Types of result** Perceptions  Experiences |
| Contandriopoulos (2012) (33)  **Study design** Systematic review | **Time frame** Cross-sectional  **Data collection** OTher  **Data analysis** Realist | **Sample population** Other  **Setting** Health care  **Discipline** Health policy | **Barriers to use of evidence** Timing/opportunity  Availability of research/access to research or info  Ambiguity/relevance/reliability of findings | **Facilitators to use of evidence** contact with researchers/info staff  Contact with policy makers  relationship with researchers/info staff  Relationship with policy-makers | **Types of evidence used** Systematic reviews  Guidelines  Primary research  Other information - not formal research |
| Currie (2011) (34)  **Study design** Case study | **Time frame** Longitudinal  **Data collection** Semi-structured interviews  Documentary analysis | **Sample population** Policy advisor  Policy-makers  Other  **Setting** Policy environment  **Discipline** drugs policy  public health |  | **Facilitators to use of evidence** importance of research findings | **Types of evidence used** Not stated |
| Deelstra (2002) (35)  **Study design** Case study | **Time frame** Cross-sectional  **Data collection** not stated  **Data analysis** Not stated | **Sample population** Policy advisor  Policy-makers  **Setting** Policy environment  **Discipline** Conservation & environmental management  transport | **Barriers to use of evidence** Political support (will)  Political support (practical)  Ambiguity/relevance/reliability of findings  Professional/international bodies  importance of policy | **Facilitators to use of evidence** relationship with researchers/info staff  Relationship with policy-makers  Collaboration  Timing/opportunity  Material resources available  Availability of research/access to research or info  Clarity/relevance/reliability of research findings  Policy-maker research awareness  Policy-maker research skills  professional bodies  Other | **Types of evidence used** Other information - not formal research  **Types of result** Experiences |
| Dionne (2006) (36)  **Study design** Qualitative study | **Time frame** Cross-sectional  **Data collection** Semi-structured interviews  **Data analysis** Thematic analysis | **Sample population** Physicians  Health care managers  **Setting** Health care  **Discipline** Primary care  Secondary & tertiary care | **Barriers to use of evidence** Staff or personnel resources  Availability of research/access to research or info |  | **Types of evidence used** Not stated  **Types of result** Perceptions  Experiences |
| Djibuti (2007) (37)  **Study design** Qualitative study | **Time frame** Cross-sectional  **Data collection** Focus Group  **Data analysis** Thematic analysis | **Sample population** Physicians  Health care managers  **Setting** Health care  **Discipline** public health | **Barriers to use of evidence** Costs  Political support (practical) |  | **Types of evidence used** Other information - not formal research  **Types of result** Behaviour  Perceptions  Experiences |
| Dobbins (2001) (38)  **Study design** Case study | **Time frame** Cross-sectional  **Data collection** Survey  **Data analysis** Descriptive statistics  Regression analysis | **Sample population** Policy-makers  Health care managers  Other  **Setting** Health care  **Discipline** public health |  | **Facilitators to use of evidence** Clarity/relevance/reliability of research findings  Policy-maker research awareness  Other | **Types of evidence used** Systematic reviews  **Types of result** Behaviour  Other |
| Dobbins (2007) (39)  **Study design** Qualitative study | **Time frame** Cross-sectional  **Data collection** Semi-structured interviews  **Data analysis** Content analysis | **Sample population** Physicians  Nurses  Policy-makers  Health care managers  **Setting** Health care  **Discipline** public health |  | **Facilitators to use of evidence** contact with researchers/info staff  Clarity/relevance/reliability of research findings  Format of research findings  Improved dissemination  importance of research findings | **Types of evidence used** Primary research  Other information - not formal research  **Types of result** Perceptions  Experiences |
| Dobbins (2009) (40)  **Study design** other | **Time frame** Cross-sectional  **Data collection** not stated  **Data analysis** Not stated | **Sample population** Policy-makers  **Setting** Policy environment  **Discipline** public health |  | **Facilitators to use of evidence** relationship with researchers/info staff  Relationship with policy-makers  Collaboration  Staff or personnel resources  Material resources available  Availability of research/access to research or info  Format of research findings  Other | **Types of evidence used** Primary research  **Types of result** Experiences |
| Dobrow (2006) (41)  **Study design** Case study | **Time frame** Cross-sectional  **Data collection** Semi-structured interviews  Documentary analysis  **Data analysis** Thematic analysis | **Sample population** Researchers  Physicians  Surgeons  Policy-makers  Other  **Setting** Health care  **Discipline** Primary care  Secondary & tertiary care  public health | **Barriers to use of evidence** Political support (will)  Practitioner research awareness  Practitioner research skills | **Facilitators to use of evidence** Political support (will)  Availability of research/access to research or info  importance of research findings | **Types of evidence used** Systematic reviews  Guidelines  Primary research  Other information - not formal research  **Types of result** Experiences |
| Dodson (2012) (42)  **Study design** Quantitative study | **Time frame** Cross-sectional  **Data collection** Survey  not stated  OTher  **Data analysis** Descriptive statistics | **Sample population** Policy advisor  Other  **Setting** Policy environment  **Discipline** public health | **Barriers to use of evidence** Availability of research/access to research or info  Ambiguity/relevance/reliability of findings  Format of research findings | **Facilitators to use of evidence** Managerial support (practical)  Improved dissemination | **Types of evidence used** Other information - not formal research  **Types of result** Other |
| Eddama (2008) (43)  **Study design** Systematic review | **Time frame** Cross-sectional  **Data collection** OTher  **Data analysis** Not stated | **Sample population** Researchers  GPs  Physicians  Allied health professionals  Policy-makers  Health care managers  **Setting** Not stated  **Discipline** Primary care  Health policy  Secondary & tertiary care | **Barriers to use of evidence** Timing/opportunity  Costs  Ambiguity/relevance/reliability of findings  Guidelines or policy statement  Other | **Facilitators to use of evidence** Policy-maker research awareness  Policy-maker research skills | **Types of evidence used** Primary research  **Types of result** Perceptions  Experiences |
| Egmond (2011) (44)  **Study design** Case study  Qualitative study | **Time frame** Cross-sectional  **Data collection** Semi-structured interviews  Documentary analysis  **Data analysis** Not stated | **Sample population** Policy-makers  **Setting** Policy environment  **Discipline** public health | **Barriers to use of evidence** Other | **Facilitators to use of evidence** Collaboration | **Types of evidence used** Systematic reviews  Primary research  **Types of result** Behaviour  Perceptions |
| El-Jardali (2012) (45)  **Study design** Quantitative study | **Time frame** Cross-sectional  **Data collection** Survey  **Data analysis** Descriptive statistics  Regression analysis | **Sample population** Researchers  **Setting** Academic institution  **Discipline** Health policy | **Barriers to use of evidence** contact with researchers/info staff  Contact with policy makers  Timing/opportunity  Managerial support (practical)  Political support (will)  Political support (practical)  Availability of research/access to research or info  Ambiguity/relevance/reliability of findings  Format of research findings | **Facilitators to use of evidence** Contact with policy makers  Managerial support (practical)  professional bodies  Improved dissemination  Other | **Types of evidence used** Primary research  **Types of result** Perceptions |
| Elliott (2000) (46)  **Study design** Case study  Qualitative study | **Time frame** Cross-sectional  **Data collection** Semi-structured interviews  Documentary analysis  Observation - written notes  **Data analysis** Not stated | **Sample population** Researchers  Policy-makers  Health care managers  Local authority staff  Other  **Setting** Health care  **Discipline** Primary care  Secondary & tertiary care | **Barriers to use of evidence** Turnover of staff  Ambiguity/relevance/reliability of findings  Policy-maker research skills  Other | **Facilitators to use of evidence** Collaboration | **Types of evidence used** Primary research  Other information - not formal research  **Types of result** Perceptions |
| Elshaug (2008) (47)  **Study design** Qualitative study | **Time frame** Cross-sectional  **Data collection** Semi-structured interviews  **Data analysis** Thematic analysis | **Sample population** Health care managers  **Setting** Health care  **Discipline** Primary care  Secondary & tertiary care | **Barriers to use of evidence** Costs  Availability of research/access to research or info  Ambiguity/relevance/reliability of findings | **Facilitators to use of evidence** Relationship with policy-makers  Costs  Political support (will)  Political support (practical)  Managerial will  Managerial support (practical) | **Types of evidence used** Not stated  **Types of result** Perceptions  Experiences |
| Ensor (2009) (48)  **Study design** Case study Qualitative study | **Time frame** Cross-sectional  **Data collection** Semi-structured interviews  **Data analysis** Not stated | **Sample population** Policy-makers  Other  **Setting** Policy environment  **Discipline** Primary care  Secondary & tertiary care | **Barriers to use of evidence** Other | **Facilitators to use of evidence** relationship with researchers/info staff  Relationship with policy-makers  Collaboration  Political support (will)  Political support (practical)  Availability of research/access to research or info  importance of research findings | **Types of evidence used** Primary research  **Types of result** Behaviour  Perceptions  Experiences |
| Ettelt (2011) (49)  **Study design** Quantitative study | **Time frame** Cross-sectional  **Data collection** Survey  **Data analysis** Descriptive statistics | **Sample population** Researchers  **Setting** Academic institution  Policy environment  **Discipline** Health policy | **Barriers to use of evidence** Timing/opportunity  Costs  Political support (will)  Political support (practical)  Availability of research/access to research or info  Ambiguity/relevance/reliability of findings  Format of research findings  Other | **Facilitators to use of evidence** Contact with policy makers  Improved dissemination  Other | **Types of evidence used** Primary research  **Types of result** Perceptions  Other |
| Feldman (2001) (50)  **Study design** Qualitative study | **Time frame** Cross-sectional  **Data collection** Semi-structured interviews  Group interviews | **Sample population** Health care managers  Information/surveillance staff  **Setting** Health care  Policy environment  **Discipline** Primary care  Secondary & tertiary care | **Barriers to use of evidence** Costs  Availability of research/access to research or info  Format of research findings  Other | **Facilitators to use of evidence** relationship with researchers/info staff  Relationship with policy-makers  Availability of research/access to research or info  Clarity/relevance/reliability of research findings  Format of research findings  professional bodies  Improved dissemination | **Types of evidence used** Primary research  **Types of result** suggestions/proposed factors  Behaviour  Perceptions  Experiences |
| Fickel (2005) (51)  **Study design** Quantitative study  Qualitative study | **Time frame** Cross-sectional  **Data collection** Survey  Semi-structured interviews  **Data analysis** Thematic analysis  Descriptive statistics | **Sample population** Health care managers  **Discipline** Health policy |  | **Facilitators to use of evidence** Availability of research/access to research or info  Clarity/relevance/reliability of research findings  Policy-maker research awareness  Policy-maker research skills | **Types of evidence used** Primary research  **Types of result** Behaviour  Experiences |
| Figgs (2000) (52)  **Study design** Quantitative study  other | **Time frame** Cross-sectional  **Data collection** Survey  **Data analysis** Descriptive statistics | **Sample population** Information/surveillance staff  **Setting** Other  **Discipline** public health | **Barriers to use of evidence** Staff or personnel resources  Availability of research/access to research or info |  | **Types of evidence used** Other information - not formal research  **Types of result** Perceptions |
| Flitcroft (2011) (53)  **Study design** Case study Qualitative study | **Time frame** Cross-sectional  **Data collection** Semi-structured interviews  **Data analysis** Thematic analysis | **Sample population** Researchers  Physicians  Surgeons  Policy advisor  Policy-makers  Health care managers  **Setting** Academic institution  Health care  Policy environment  **Discipline** Primary care  Secondary & tertiary care | **Barriers to use of evidence** Contact with policy makers  relationship with researchers/info staff  Relationship with policy-makers  Timing/opportunity  Turnover of staff  Costs  Managerial support (practical)  Other |  | **Types of evidence used** Systematic reviews  Guidelines  Primary research  **Types of result** Perceptions  Experiences |
| Fournier (2012) (54)  **Study design** Case study | **Time frame** Cross-sectional  **Data collection** Semi-structured interviews  Documentary analysis  **Data analysis** Thematic analysis | **Sample population** Researchers  Other  **Setting** Health care  **Discipline** Primary care  Secondary & tertiary care | **Barriers to use of evidence** Availability of research/access to research or info | **Facilitators to use of evidence** relationship with researchers/info staff  Political support (will)  Managerial support (practical)  Improved dissemination  Other | **Types of evidence used** Systematic reviews  Guidelines Primary research Other information - not formal research  **Types of result** Documentary proof of research use  Perceptions |
| Frey (2011) (55)  **Study design** Case study | **Time frame** Cross-sectional  **Data collection** Semi-structured interviews  Documentary analysis  **Data analysis** Thematic analysis  Content analysis | **Sample population** Policy advisor  Policy-makers  Other  **Setting** Policy environment  **Discipline** Other  drugs policy  transport | **Barriers to use of evidence** Consumer-related barrier |  | **Types of evidence used** Primary research  **Types of result** Documentary proof of research use |
| Friese (2009) (56)  **Study design** Qualitative study | **Time frame** Cross-sectional  **Data collection** Semi-structured interviews  **Data analysis** Thematic analysis | **Sample population** Researchers  **Setting** Policy environment  **Discipline** Health policy  Education  social care / social work | **Barriers to use of evidence** Relationship with policy-makers **Barriers to use of evidence** Policy-maker research skills **Barriers to use of evidence** Policy-maker research awareness | **Facilitators to use of evidence** Contact with policy makers  Relationship with policy-makers  Timing/opportunity  Clarity/relevance/reliability of research findings  Format of research findings  Policy-maker research skills  Other | **Types of evidence used** Primary research  **Types of result** Behaviour  Perceptions  Experiences |
| Gagliardi (2008) (57)  **Study design** Quantitative study  Case study  Qualitative study | **Time frame** Cross-sectional  **Data collection** Survey  Semi-structured interviews  **Data analysis** Thematic analysis  Descriptive statistics | **Sample population** Researchers  GPs  Physicians  Surgeons  Nurses  Allied health professionals  Policy-makers  Health care managers  **Setting** Health care  **Discipline** Primary care  Secondary & tertiary care |  | **Facilitators to use of evidence** contact with researchers/info staff  Contact with policy makers  relationship with researchers/info staff  Relationship with policy-makers  Collaboration  Timing/opportunity | **Types of evidence used** Primary research  **Types of result** Perceptions |
| Galani (2008) (58)  **Study design** Systematic review | **Time frame** Cross-sectional  **Data collection** OTher  **Data analysis** Descriptive - no analysis | **Sample population** Other  **Setting** Not applicable  **Discipline** Primary care  Secondary & tertiary care | **Barriers to use of evidence** Timing/opportunity  Availability of research/access to research or info  Ambiguity/relevance/reliability of findings  Policy-maker research skills  Policy-maker research awareness  Other | **Facilitators to use of evidence** Policy-maker research skills  Improved dissemination  Guidelines or policy statement | **Types of evidence used** Primary research  **Types of result** Other |
| Garrib (2008) (59)  **Study design** Qualitative study | **Time frame** Cross-sectional  **Data collection** Semi-structured interviews  **Data analysis** Descriptive statistics | **Sample population** Health care managers  Information/surveillance staff  **Setting** Health care  **Discipline** Primary care  Secondary & tertiary care | **Barriers to use of evidence** Timing/opportunity  Costs  Staff or personnel resources  Lack of material resources  Practitioner research skills | **Facilitators to use of evidence** Practitioner research skills | **Types of evidence used** Other information - not formal research  **Types of result** Other |
| Gkeredakis (2011) (60)  **Study design** Qualitative study | **Time frame** Cross-sectional  **Data collection** Semi-structured interviews  Documentary analysis  Observation - written notes  **Data analysis** Other | **Sample population** Health care managers  **Setting** Health care  **Discipline** Health policy | **Barriers to use of evidence** relationship with researchers/info staff  Relationship with policy-makers  Managerial support (practical)  Ambiguity/relevance/reliability of findings |  | **Types of evidence used** Not stated  **Types of result** Perceptions |
| Goede (2011) (61)  **Study design** Case study | **Time frame** Cross-sectional  **Data collection** Semi-structured interviews  Documentary analysis  **Data analysis** Thematic analysis  Descriptive statistics | **Sample population** Researchers  Health care managers  Local authority staff  **Setting** Policy environment  **Discipline** public health | **Barriers to use of evidence** Ambiguity/relevance/reliability of findings | **Facilitators to use of evidence** Relationship with policy-makers  Collaboration  Political support (will)  Clarity/relevance/reliability of research findings  Format of research findings | **Types of evidence used** Other information - not formal research  **Types of result** Perceptions |
| GB National Audit Office (2003) (62)  **Study design** Case study  Qualitative study  other | **Time frame** Cross-sectional  **Data collection** Survey  Semi-structured interviews  Documentary analysis  **Data analysis** Not stated | **Sample population** Researchers  Policy advisor  Other  **Setting** Policy environment  **Discipline** Conservation & environmental management  social care / social work | **Barriers to use of evidence** relationship with researchers/info staff  Relationship with policy-makers  Timing/opportunity  Lack of material resources  Availability of research/access to research or info  Ambiguity/relevance/reliability of findings  Policy-maker research skills  Policy-maker research awareness | **Facilitators to use of evidence** relationship with researchers/info staff  Relationship with policy-makers  Collaboration  Political support (practical)  Policy-maker research skills  professional bodies  Improved dissemination  Other | **Types of evidence used** Systematic reviews  Primary research Other information - not formal research |
| Green (2011) (63)  **Study design** Case study  Qualitative study | **Time frame** Cross-sectional  **Data collection** Semi-structured interviews  Focus Group  Group interviews  Documentary analysis  **Data analysis** Other | **Sample population** Midwives  Policy-makers  **Setting** Health care  Policy environment  **Discipline** Other |  | **Facilitators to use of evidence** Managerial support (practical)  Clarity/relevance/reliability of research findings  Legal support  Other | **Types of evidence used** Primary research  Other information - not formal research  **Types of result** Perceptions |
| Greyson (2012) (64)  **Study design** Quantitative study | **Time frame** Cross-sectional  **Data collection** Semi-structured interviews  **Data analysis** Thematic analysis | **Sample population** Policy-makers  **Setting** Policy environment  **Discipline** drugs policy | **Barriers to use of evidence** Timing/opportunity  Availability of research/access to research or info  Format of research findings  Other |  | **Types of evidence used** Not stated  **Types of result** Perceptions |
| Hamel (2011) (65)  **Study design** Case study | **Time frame** Cross-sectional  **Data collection** Semi-structured interviews  Documentary analysis  **Data analysis** Thematic analysis | **Sample population** Health care managers  Other  **Setting** Other  **Discipline** public health |  | **Facilitators to use of evidence** contact with researchers/info staff  Contact with policy makers  Collaboration  Costs  Staff or personnel resources  Material resources available  Political support (will)  Managerial will  Managerial support (practical)  Availability of research/access to research or info  Clarity/relevance/reliability of research findings  Policy-maker research skills  Improved dissemination  Other  importance of policy  importance of research findings | **Types of evidence used** Primary research  **Types of result** Documentary proof of research use  Perceptions |
| Haynes (2011) (66)  **Study design** Qualitative study | **Time frame** Cross-sectional  **Data collection** Survey  **Data analysis** Thematic analysis | **Sample population** Policy advisor  Policy-makers  **Setting** Policy environment  **Discipline** public health |  | **Facilitators to use of evidence** contact with researchers/info staff  Contact with policy makers  relationship with researchers/info staff  Relationship with policy-makers  Managerial support (practical)  Improved dissemination  Other  importance of policy | **Types of evidence used** Other information - not formal research |
| Haynes (2011) (67)  **Study design** Qualitative study | **Time frame** Cross-sectional  **Data collection** Survey  Semi-structured interviews  **Data analysis** Thematic analysis | **Sample population** Researchers  **Setting** Academic institution  **Discipline** public health | **Barriers to use of evidence** Managerial support (practical) | **Facilitators to use of evidence** Contact with policy makers  Relationship with policy-makers  Collaboration  Availability of research/access to research or info  Clarity/relevance/reliability of research findings  Improved dissemination  Other | **Types of evidence used** Not stated  **Types of result** Perceptions  Experiences |
| Henderson (2009) (68)  **Study design** Quantitative study | **Time frame** Cross-sectional  **Data collection** Survey  **Data analysis** Regression analysis | **Sample population** Policy-makers  Other  **Setting** Other  **Discipline** criminal justice |  | **Facilitators to use of evidence** Collaboration  Staff or personnel resources  Material resources available  Managerial will  Managerial support (practical)  Other | **Types of evidence used** Systematic reviews  Primary research  **Types of result** Perceptions |
| Hennink (2005) (69)  **Study design** Case study  Qualitative study | **Time frame** Cross-sectional  **Data collection** Semi-structured interviews  **Data analysis** Thematic analysis | **Sample population** Researchers  Policy-makers  Health care managers  **Setting** Policy environment  **Discipline** Health policy | **Barriers to use of evidence** contact with researchers/info staff  Contact with policy makers  Collaboration  Costs  Political support (will)  Lack of material resources  Availability of research/access to research or info  Ambiguity/relevance/reliability of findings  Format of research findings  Policy-maker research skills  Policy-maker research awareness  Other | **Facilitators to use of evidence** contact with researchers/info staff  Contact with policy makers  relationship with researchers/info staff  Relationship with policy-makers  professional bodies  Improved dissemination | **Types of evidence used** Primary research  Other information - not formal research  **Types of result** Perceptions  Experiences |
| Higgins (2011) (70)  **Study design** Qualitative study | **Time frame** Cross-sectional  **Data collection** Semi-structured interviews  **Data analysis** Thematic analysis | **Sample population** Other  **Setting** Health care  **Discipline** public health | **Barriers to use of evidence** Availability of research/access to research or info  Format of research findings  Other | **Facilitators to use of evidence** Availability of research/access to research or info | **Types of evidence used** Other information - not formal research  **Types of result** Perceptions |
| Hinchcliff (2011) (71)  **Study design** Case study  Qualitative study | **Time frame** Longitudinal  **Data collection** Semi-structured interviews  **Data analysis** Thematic analysis | **Sample population** Researchers  Policy-makers  Other  Legal staff  **Setting** Policy environment  **Discipline** transport  public health |  | **Facilitators to use of evidence** Political support (will)  importance of policy | **Types of evidence used** Primary research  Other information - not formal research  **Types of result** Perceptions |
| Hinchcliff (2010) (72)  **Study design** Qualitative study | T**ime frame** Cross-sectional  **Data collection** Semi-structured interviews  **Data analysis** Thematic analysis | **Sample population** Policy advisor  Policy-makers  Local authority staff  Other  **Setting** Policy environment  **Discipline** transport  public health | **Barriers to use of evidence** Costs  Availability of research/access to research or info  Ambiguity/relevance/reliability of findings  Other | **Facilitators to use of evidence** relationship with researchers/info staff  Relationship with policy-makers  Costs  Political support (will)  Political support (practical)  Availability of research/access to research or info  Clarity/relevance/reliability of research findings  Improved dissemination  Other | **Types of evidence used** Primary research  **Types of result** Perceptions |
| Hird (2005) (73)  **Study design** Quantitative study | **Time frame** Cross-sectional  **Data collection** Survey  **Data analysis** Descriptive statistics | **Sample population** Policy-makers  **Setting** Policy environment  **Discipline** Other |  | **Facilitators to use of evidence** Relationship with policy-makers  Availability of research/access to research or info  Clarity/relevance/reliability of research findings  Improved dissemination  Other | **Types of evidence used** Primary research  Other information - not formal research  **Types of result** Perceptions  Experiences |
| Hivon (2005)(74)  **Study design** Qualitative study | **Time frame** Cross-sectional  **Data collection** Semi-structured interviews  **Data analysis** Thematic analysis | **Sample population** Physicians  Health care managers  Other  **Setting** Policy environment  **Discipline** Primary care  Secondary & tertiary care | **Barriers to use of evidence** Timing/opportunity  Costs  Staff or personnel resources  Managerial support (practical)  Political support (will)  Availability of research/access to research or info  Practitioner research skills  Other |  | **Types of evidence used** Primary research  **Types of result** Perceptions  Experiences |
| Hobin (2012) (75)  **Study design** Qualitative study | **Time frame** Cross-sectional  **Data collection** Focus Group  Documentary analysis  **Data analysis** Not stated | **Sample population** Researchers  Policy-makers  **Setting** Not stated  **Discipline** public health | **Barriers to use of evidence** Availability of research/access to research or info  Ambiguity/relevance/reliability of findings | **Facilitators to use of evidence** Collaboration  Clarity/relevance/reliability of research findings | **Types of evidence used** Systematic reviews  Primary research  **Types of result** suggestions/proposed factors  Perceptions |
| Hooton (2010) (76)  **Study design** Case study | **Time frame** Longitudinal  **Data collection** Semi-structured interviews  Focus Group  Documentary analysis  **Data analysis** Thematic analysis  Other | **Sample population** Policy-makers  **Setting** Policy environment  **Discipline** Other |  | **Facilitators to use of evidence** Collaboration  Political support (will)  Clarity/relevance/reliability of research findings  Improved dissemination | **Types of evidence used** Systematic reviews  Primary research  **Types of result** Perceptions |
| Hunsmann M. (2012) (77)  **Study design** Qualitative study | **Time frame** Cross-sectional  **Data collection** Semi-structured interviews  Observation - written notes  **Data analysis** Thematic analysis | **Sample population** Researchers  Policy advisor  Policy-makers  Other  **Setting** Policy environment  **Discipline** public health | **Barriers to use of evidence** Timing/opportunity  Ambiguity/relevance/reliability of findings  Policy-maker research awareness  Other |  | **Types of evidence used** Primary research  **Types of result** Perceptions |
| Hutchinson (2011) (78)  **Study design** Case study | **Time frame** Longitudinal  **Data collection** Semi-structured interviews Documentary analysis  **Data analysis** Thematic analysis | **Sample population** Researchers  Physicians  Policy advisor  Policy-makers  Other  **Setting** Health care  Policy environment  **Discipline** drugs policy | **Barriers to use of evidence** Lack of material resources  Ambiguity/relevance/reliability of findings  Other  Professional/international bodies  importance of policy | **Facilitators to use of evidence** Costs | **Types of evidence used** Primary research  **Types of result** Behaviour  Perceptions |
| Hyder (2011) (79)  **Study design** Qualitative study | **Time frame** Cross-sectional  **Data collection** Semi-structured interviews  **Data analysis** Thematic analysis | **Sample population** Policy advisor  Policy-makers  **Setting** Health care  Policy environment  **Discipline** Health policy | **Barriers to use of evidence** Availability of research/access to research or info  Policy-maker research skills  Policy-maker research awareness  Other  Professional/international bodies | **Facilitators to use of evidence** relationship with researchers/info staff  Format of research findings  Policy-maker research skills  Improved dissemination | **Types of evidence used** Primary research  **Types of result** Perceptions |
| Innvaer (2009)  (80)  **Study design** other | **Time frame** Cross-sectional  **Data collection** Documentary analysis  **Data analysis** Descriptive statistics  Other | **Sample population** Other  **Setting** Policy environment  **Discipline** Primary care  Other  drugs policy  Secondary & tertiary care  public health | **Barriers to use of evidence** contact with researchers/info staff | **Facilitators to use of evidence** Clarity/relevance/reliability of research findings  Other  importance of research findings | **Types of evidence used** Primary research  **Types of result** Documentary proof of research use |
| Innvaer (2002) (81)  **Study design** Systematic review | **Time frame** Cross-sectional  **Data collection** OTher  **Data analysis** Descriptive - no analysis | **Sample population** Policy-makers  Health care managers  **Setting** Not applicable  **Discipline** Health policy | **Barriers to use of evidence** contact with researchers/info staff  relationship with researchers/info staff  Timing/opportunity  Turnover of staff  Costs  Ambiguity/relevance/reliability of findings  Policy-maker research skills  Other | **Facilitators to use of evidence** contact with researchers/info staff  Timing/opportunity  Clarity/relevance/reliability of research findings  Format of research findings  Other  importance of policy | **Types of evidence used** Not stated  **Types of result** Behaviour  Perceptions  Intentions  Experiences |
| Jack (2010) (82)  **Study design** Qualitative study | **Time frame** Cross-sectional  **Data collection** Semi-structured interviews  **Data analysis** Content analysis | **Sample population** Researchers  Policy-makers  Other  **Setting** Other  **Discipline** health promotion  Other  public health | **Barriers to use of evidence** Costs  Availability of research/access to research or info  Consumer-related barrier | **Facilitators to use of evidence** relationship with researchers/info staff  Collaboration  Clarity/relevance/reliability of research findings  Improved dissemination  Other | **Types of evidence used** Primary research  Other information - not formal research  **Types of result** Perceptions  Experiences |
| Jacobs (2010)  (83)  **Study design** Quantitative study Qualitative study | **Time frame** Cross-sectional  **Data collection** Survey  Semi-structured interviews  **Data analysis** Thematic analysis  Descriptive statistics | **Sample population** Researchers  Policy-makers  Health care managers  Information/surveillance staff  **Setting** Health care  **Discipline** Secondary & tertiary care | **Barriers to use of evidence** Contact with policy makers  Managerial support (practical)  Lack of material resources  Ambiguity/relevance/reliability of findings | **Facilitators to use of evidence** Legal support | **Types of evidence used** Other information - not formal research  **Types of result** Documentary proof of research use  Perceptions |
| Jbilou (2007) (84)  **Study design** Quantitative study | **Time frame** Cross-sectional  **Data collection** Survey  **Data analysis** Descriptive statistics  Regression analysis | **Sample population** Policy-makers  Health care managers  **Setting** Policy environment  **Discipline** Primary care  Secondary & tertiary care |  | **Facilitators to use of evidence** relationship with researchers/info staff  Collaboration  Material resources available  Clarity/relevance/reliability of research findings  Other | **Types of evidence used** Primary research  **Types of result** Behaviour  Other |
| Jenkins (2005) (85)  **Study design** Case study | **Time frame** Cross-sectional  **Data collection** Semi-structured interviews  **Data analysis** Thematic analysis | **Sample population** Other  **Setting** Health care  **Discipline** public health | **Barriers to use of evidence** Availability of research/access to research or info  Format of research findings  Policy-maker research skills  Practitioner research skills  Other | **Facilitators to use of evidence** contact with researchers/info staff  Relationship with policy-makers  Collaboration  Material resources available  Managerial support (practical)  Availability of research/access to research or info  Policy-maker research skills  Other | **Types of evidence used** Primary research  Other information - not formal research  **Types of result** suggestions/proposed factors  Behaviour  Perceptions  Experiences |
| Jennings (2012) (86)  **Study design** Quantitative study | **Time frame** Cross-sectional  **Data collection** Survey  **Data analysis** Descriptive statistics  Regression analysis | **Sample population** Policy advisor  Policy-makers  **Setting** Policy environment  **Discipline** Primary care  health promotion  criminal justice  Other  Conservation & environmental management  drugs policy  social care / social work  transport  public health |  | **Facilitators to use of evidence** Availability of research/access to research or info | **Types of evidence used** Primary research **Types of evidence used** Other information - not formal research  **Types of result** Perceptions |
| Jewell (2008)  (87)  **Study design** Case study | **Time frame** Cross-sectional  **Data collection** Semi-structured interviews  **Data analysis** Thematic analysis | **Sample population** Policy-makers  Health care managers  **Setting** Policy environment  **Discipline** Health policy | **Barriers to use of evidence** contact with researchers/info staff  Contact with policy makers  Relationship with policy-makers  Timing/opportunity  Turnover of staff  Costs  Staff or personnel resources  Availability of research/access to research or info  Ambiguity/relevance/reliability of findings  Lack of legal support  Consumer-related barrier  Policy-maker research skills  Policy-maker research awareness  Practitioner research skills  Other  Professional/international bodies  importance of policy | **Facilitators to use of evidence** relationship with researchers/info staff  Collaboration  Timing/opportunity  Managerial support (practical)  Availability of research/access to research or info  Clarity/relevance/reliability of research findings  Format of research findings  Policy-maker research skills  Improved dissemination | **Types of evidence used** Systematic reviews  Guidelines  Primary research  Other information - not formal research  **Types of result** Perceptions  Experiences |
| Jonsson (2005) (88)  **Study design** Quantitative study  Qualitative study | **Time frame** Cross-sectional  **Data collection** Survey  Semi-structured interviews  Group interviews  **Data analysis** Descriptive statistics  Not stated | **Sample population** Researchers  Physicians  Nurses  Allied health professionals  Policy-makers  Health care managers  Other  **Setting** Policy environment  **Discipline** Health policy | **Barriers to use of evidence** Collaboration  Costs  Availability of research/access to research or info  Policy-maker research awareness  Other | **Facilitators to use of evidence** Contact with policy makers  Collaboration  Availability of research/access to research or info  Policy-maker research skills  professional bodies  Improved dissemination  Other | **Types of evidence used** Primary research  **Types of result** Perceptions  Experiences |
| Kapiriri (2006) (89)  **Study design** Quantitative study | **Time frame** Cross-sectional  **Data collection** Survey  **Data analysis** Descriptive statistics  Regression analysis | **Sample population** Physicians  Nurses  Health care managers  Local authority staff  **Setting** Health care  **Discipline** Primary care  Secondary & tertiary care | **Barriers to use of evidence** Availability of research/access to research or info  Other | **Facilitators to use of evidence** Relationship with policy-makers  Guidelines or policy statement | **Types of evidence used** Primary research  Other information - not formal research  **Types of result** suggestions/proposed factors  Behaviour  Perceptions  Experiences |
| Kok (2012) (90)  **Study design** Qualitative study | **time frame** Cross-sectional  **Data collection** Semi-structured interviews  **Data analysis** Thematic analysis | **Sample population** Other  **Setting** Health care  Policy environment  **Discipline** health promotion | **Barriers to use of evidence** Timing/opportunity  Political support (will)  Availability of research/access to research or info | **Facilitators to use of evidence** contact with researchers/info staff  relationship with researchers/info staff  Availability of research/access to research or info  Improved dissemination | **Types of evidence used** Primary research  **Types of result** Perceptions |
| Kok (2012) (91)  **Study design** Case study | **Time frame** Longitudinal  **Data collection** Semi-structured interviews  Documentary analysis  **Data analysis** Thematic analysis | **Sample population** Researchers  Policy advisor  Policy-makers  **Setting** Academic institution  Health care  **Discipline** Health policy | **Barriers to use of evidence** Availability of research/access to research or info  Professional/international bodies  importance of research findings | **Facilitators to use of evidence** contact with researchers/info staff  Contact with policy makers | **Types of evidence used** Primary research  **Types of result** Perceptions |
| Kothari (2004)  (92)  **Study design** Case study | T**ime frame** Cross-sectional  **Data collection** Semi-structured interviews  Group interviews  **Data analysis** Thematic analysis | **Sample population** Health care managers  Other  **Setting** Health care  **Discipline** Primary care  Secondary & tertiary care  public health |  | **Facilitators to use of evidence** Collaboration | **Types of evidence used** Guidelines  **Types of result** suggestions/proposed factors **Types of result** Behaviour  Perceptions  Experiences |
| Kothari (2009)  (93)  **Study design** Qualitative study | **Time frame** Cross-sectional  **Data collection** Survey  Focus Group  **Data analysis** Thematic analysis  Descriptive statistics | **Sample population** Policy-makers  Health care managers  **Setting** Health care  **Discipline** Primary care  Health policy  Secondary & tertiary care | **Barriers to use of evidence** Timing/opportunity  Costs  Availability of research/access to research or info  Ambiguity/relevance/reliability of findings  Policy-maker research skills  Other | **Facilitators to use of evidence** contact with researchers/info staff  relationship with researchers/info staff  Staff or personnel resources | **Types of evidence used** Not stated  **Types of result** Behaviour  Perceptions  Experiences |
| Kurko (2012) (94)  **Study design** Case study | **Time frame** Longitudinal  **Data collection** Documentary analysis  **Data analysis** Content analysis | **Sample population** Other  **Setting** Policy environment  **Discipline** drugs policy | **Barriers to use of evidence** importance of research findings |  | **Types of evidence used** Primary research  Other information - not formal research  **Types of result** Documentary proof of research use |
| Lairumbi (2008) (95)  **Study design** Qualitative study | **Time frame** Cross-sectional  **Data collection** Semi-structured interviews  **Data analysis** Thematic analysis | **Sample population** Researchers  Policy-makers  Other  **Setting** Health care  Policy environment  Other  **Discipline** Health policy | **Barriers to use of evidence** contact with researchers/info staff  Turnover of staff  Staff or personnel resources  Lack of material resources  Availability of research/access to research or info  Policy-maker research awareness  Other | **Facilitators to use of evidence** Collaboration  Improved dissemination | **Types of evidence used** Primary research  **Types of result** Perceptions |
| Larsen (2012) (96)  **Study design** Quantitative study | **Time frame** Cross-sectional  **Data collection** Survey  **Data analysis** Descriptive statistics  Regression analysis | **Sample population** Health care managers  **Setting** Health care  **Discipline** Primary care  Secondary & tertiary care  public health |  | **Facilitators to use of evidence** Political support (will)  Managerial support (practical)  Policy-maker research skills  Other | **Types of evidence used** Systematic reviews  Guidelines  Primary research  Other information - not formal research  **Types of result** Perceptions  Intentions |
| Lavis (2001) (97)  **Study design** Qualitative study | **Time frame** Cross-sectional  **Data collection** Survey  Semi-structured interviews  **Data analysis** Thematic analysis | **Sample population** Policy-makers  Other  **Setting** Policy environment  **Discipline** Other  public health | **Barriers to use of evidence** Availability of research/access to research or info  Ambiguity/relevance/reliability of findings  Lack of legal support  Consumer-related barrier  importance of research findings |  | **Types of evidence used** Other information - not formal research  **Types of result** Perceptions  Experiences |
| Learmonth (2000) (98)  **Study design** Case study | **Time frame** Cross-sectional  **Data collection** OTher | **Sample population** Policy-makers  Local authority staff  **Setting** Policy environment  **Discipline** health promotion | **Barriers to use of evidence** Costs  Other | **Facilitators to use of evidence** relationship with researchers/info staff  Collaboration  Guidelines or policy statement | **Types of evidence used** Primary research  Other information - not formal research  **Types of result** Perceptions |
| Lemieux-Charles (2002) (99)  **Study design** Case study | **Time frame** Longitudinal  **Data collection** Survey  Semi-structured interviews  Documentary analysis  **Data analysis** Not stated | **Sample population** Researchers  Policy-makers  Health care managers  **Setting** Health care  **Discipline** Secondary & tertiary care |  | **Facilitators to use of evidence** contact with researchers/info staff  Contact with policy makers  relationship with researchers/info staff  Relationship with policy-makers  Collaboration  Timing/opportunity  Costs  Political support (practical)  Availability of research/access to research or info  Practitioner research awareness  professional bodies  Guidelines or policy statement  Other | **Types of evidence used** Systematic reviews  Primary research  **Types of result** Documentary proof of research use  Other |
| Lenchucha (2010) (100)  **Study design** Systematic review | **Time frame** Cross-sectional  **Data collection** OTher  **Data analysis** Thematic analysis | **Sample population** Other  **Setting** Health care  **Discipline** Health policy | **Barriers to use of evidence** Timing/opportunity  Other | **Facilitators to use of evidence** relationship with researchers/info staff  Relationship with policy-makers  Collaboration  Costs  Staff or personnel resources  Material resources available  Practitioner research skills  Policy-maker research skills  Other | **Types of evidence used** Not stated  **Types of result** Other |
| Lewig (2010) (101)  **Study design** Case study  Qualitative study | **Time frame** Cross-sectional  **Data collection** Semi-structured interviews  Documentary analysis  **Data analysis** Thematic analysis | **Sample population** Researchers  Policy advisor  Policy-makers  Other  **Setting** Policy environment  **Discipline** Other | **Barriers to use of evidence** Collaboration  Timing/opportunity  Costs  Staff or personnel resources  Managerial support (practical)  Political support (will)  Lack of material resources  Availability of research/access to research or info  Ambiguity/relevance/reliability of findings  Policy-maker research skills  Other | **Facilitators to use of evidence** relationship with researchers/info staff  Relationship with policy-makers  Managerial support (practical)  Availability of research/access to research or info  Clarity/relevance/reliability of research findings  Policy-maker research awareness  Policy-maker research skills  Improved dissemination | **Types of evidence used** Guidelines  Primary research  Other information - not formal research  **Types of result** Documentary proof of research use  Perceptions  Experiences |
| Lomas (2009) (102)  **Study design** Case study  Qualitative study | **Time frame** Cross-sectional  **Data collection** Semi-structured interviews  **Data analysis** Thematic analysis | **Sample population** Policy advisor  Policy-makers  **Setting** Policy environment  **Discipline** Health policy | **Barriers to use of evidence** Policy-maker research awareness | **Facilitators to use of evidence** CollaborationPolicy-maker research skills  Other | **Types of evidence used** Systematic reviews  Guidelines  Primary research  Other information - not formal research  **Types of result** suggestions/proposed factors  Behaviour  Perceptions  Experiences |
| Martin (2011) (103)  **Study design** Qualitative study | **Time frame** Cross-sectional  **Data collection** Semi-structured interviews  **Data analysis** Other | **Sample population** Researchers  Policy-makers  Health care managers  **Setting** Academic institution  Policy environment  **Discipline** Primary care  Secondary & tertiary care | **Barriers to use of evidence** contact with researchers/info staff  Timing/opportunity  Turnover of staff  Political support (will)  Availability of research/access to research or info  Ambiguity/relevance/reliability of findings  Other  importance of policy  importance of research findings | **Facilitators to use of evidence** relationship with researchers/info staff  Relationship with policy-makers  Staff or personnel resources  Material resources available  Policy-maker research skills  Improved dissemination | **Types of evidence used** Primary research  **Types of result** Perceptions  Experiences |
| McAneney (2010) (104)  **Study design** Quantitative study | **Time frame** Cross-sectional  **Data collection** Survey  **Data analysis** Descriptive statistics  Other | **Sample population** Researchers  GPs  Policy-makers  Health care managers  **Setting** Academic institution  **Discipline** public health |  | **Facilitators to use of evidence** contact with researchers/info staff  Contact with policy makers  Collaboration | **Types of evidence used** Not stated  **Types of result** Perceptions |
| McBride (2008) (105)  **Study design** Qualitative study | **Time frame** Cross-sectional  **Data collection** Focus Group  **Data analysis** Thematic analysis | **Sample population** Policy advisor  Policy-makers  **Setting** Policy environment  **Discipline** Health policy | **Barriers to use of evidence** Turnover of staff  Availability of research/access to research or info  Ambiguity/relevance/reliability of findings | **Facilitators to use of evidence** contact with researchers/info staff  Contact with policy makers  relationship with researchers/info staff  Relationship with policy-makers  Timing/opportunity  Costs  Clarity/relevance/reliability of research findings  Format of research findings  Policy-maker research awareness  Policy-maker research skills  Improved dissemination  Guidelines or policy statement | **Types of evidence used** Not stated  **Types of result** suggestions/proposed factors  Behaviour  Experiences |
| McDavid (2012) (106)  **Study design** Quantitative study | **Time frame** Cross-sectional  **Data collection** Survey  **Data analysis** Descriptive statistics | **Sample population** Policy-makers  **Setting** Policy environment  **Discipline** Other | **Barriers to use of evidence** Consumer-related barrier |  | **Types of evidence used** Other information - not formal research  **Types of result** Perceptions |
| McLaughlin (2010) (107)  **Study design** Qualitative study | **Time frame** Cross-sectional  **Data collection** Semi-structured interviews  **Data analysis** Thematic analysis | **Sample population** Nurses  Allied health professionals  Policy-makers  Health care managers  Local authority staff  **Setting** Other  **Discipline** social care / social work | **Barriers to use of evidence** Timing/opportunity  Lack of material resources  Availability of research/access to research or info  Ambiguity/relevance/reliability of findings  Other | **Facilitators to use of evidence** Staff or personnel resources  Material resources available  Availability of research/access to research or info  Clarity/relevance/reliability of research findings  Format of research findings  Improved dissemination  Other | **Types of evidence used** Guidelines  Primary research  Other information - not formal research  **Types of result** Perceptions  Intentions  Experiences |
| Mercer (2010) (108)  **Study design** Case study | **Time frame** Cross-sectional  **Data collection** Documentary analysis  OTher  **Data analysis** Not stated | **Sample population** Other  **Setting** Policy environment  **Discipline** transport  public health |  | **Facilitators to use of evidence** Relationship with policy-makers  Collaboration  Timing/opportunity  Political support (practical)  Improved dissemination  Other  importance of policy  importance of research findings | **Types of evidence used** Systematic reviews  **Types of result** Behaviour  Experiences |
| Mitton (2004) (109)  **Study design** Case study other | **Time frame** Longitudinal  **Data collection** Semi-structured interviews  Focus Group  Documentary analysis  Observation - written notes  **Data analysis** Thematic analysis | **Sample population** Physicians  Policy-makers  Health care managers  **Setting** Health care  **Discipline** Primary care  Secondary & tertiary care | **Barriers to use of evidence** Timing/opportunity  Lack of material resources  Availability of research/access to research or info  Ambiguity/relevance/reliability of findings  Policy-maker research awareness  Practitioner research skills  Other | **Facilitators to use of evidence** Collaboration  Political support (will)  Managerial will  Managerial support (practical)  Other | **Types of evidence used** Primary research  Other information - not formal research  **Types of result** Behaviour  Perceptions  Experiences |
| Moore (2011) (110)  **Study design** Systematic revew | **Time frame** Cross-sectional  **Data collection** OTher  **Data analysis** Thematic analysis | **Sample population** Other  **Setting** Health care  Policy environment  **Discipline** Health policy  public health |  | **Facilitators to use of evidence** contact with researchers/info staff  Managerial support (practical)  Availability of research/access to research or info | **Types of evidence used** Not stated  **Types of result** Other |
| Murthy (2012) (111)  **Study design** Systematic review | **Time frame** Cross-sectional  **Data collection** OTher  **Data analysis** Other | **Sample population** Other  **Setting** Health care  **Discipline** Primary care  Secondary & tertiary care |  | **Facilitators to use of evidence** Contact with policy makers  Improved dissemination | **Types of evidence used** Systematic reviews  **Types of result** Other |
| Niedzwiedzka (2003) (112)  **Study design** Quantitative study  Qualitative study  other | **Time frame** Cross-sectional  **Data collection** Survey  Semi-structured interviews  Focus Group  Documentary analysis  **Data analysis** Content analysis | **Sample population** Nurses  Policy advisor  Policy-makers  Health care managers  **Setting** Policy environment  **Discipline** Primary care  Secondary & tertiary care | **Barriers to use of evidence** Timing/opportunity  Costs  Managerial support (practical)  Lack of material resources  Availability of research/access to research or info  Policy-maker research awareness  Practitioner research skills  Other  Professional/international bodies | **Facilitators to use of evidence** Collaboration | **Types of evidence used** Not stated  **Types of result** suggestions/proposed factors  Behaviour  Perceptions  Experiences |
| Norton (2012) (113)  **Study design** Quantitative study | **Time frame** Cross-sectional  **Data collection** Survey  **Data analysis** Descriptive statistics | **Sample population** Health care managers  **Setting**  Policy environment  **Discipline** public health |  | **Facilitators to use of evidence** Political support (practical)  Managerial will | **Types of evidence used** Other information - not formal research  **Types of result** Perceptions **Types of result** Intentions |
| Olson (2003) (114)  **Study design** Quantitative study | **Time frame** Cross-sectional  **Data collection** Survey  **Data analysis** Descriptive statistics | **Sample population** Researchers  Health care managers  **Setting** Health care  **Discipline** Primary care  Secondary & tertiary care | **Barriers to use of evidence** Availability of research/access to research or info  Ambiguity/relevance/reliability of findings  Policy-maker research skills  Practitioner research skills  Guidelines or policy statement | **Facilitators to use of evidence** Collaboration  Availability of research/access to research or info  Clarity/relevance/reliability of research findings  Practitioner research skills  Other | **Types of evidence used** Other information - not formal research  **Types of result** Perceptions  Experiences |
| Orem (2012) (115)  **Study design** Case study | **Time frame** Cross-sectional  **Data collection** Semi-structured interviews  Documentary analysis  **Data analysis** Thematic analysis | **Sample population** Researchers  Policy-makers  **Setting** Policy environment  **Discipline** Health policy |  | **Facilitators to use of evidence** contact with researchers/info staff  Contact with policy makers  Collaboration  Costs  Staff or personnel resources  Material resources available  Political support (will)  Political support (practical)  Availability of research/access to research or info  Format of research findings  professional bodies  Improved dissemination  Other | **Types of evidence used** Not stated  **Types of result** Perceptions |
| Ortega-Argueta (2011) (116)  **Study design** Case study | **Time frame** Cross-sectional  **Data collection** Documentary analysis  **Data analysis** Content analysis  Descriptive statistics | **Sample population** Other  **Setting** Policy environment  **Discipline** Conservation & environmental management | **Barriers to use of evidence** Availability of research/access to research or info |  | **Types of evidence used** Other information - not formal research  **Types of result** Documentary proof of research use |
| Orton (2011) (117)  **Study design** Systematic review | **Time frame** Cross-sectional  **Data collection** not stated  **Data analysis** Descriptive - no analysis | **Setting** Not applicable  **Discipline** public health | **Barriers to use of evidence** contact with researchers/info staff  Contact with policy makers  Timing/opportunity  Availability of research/access to research or info  Ambiguity/relevance/reliability of findings  Format of research findings  Policy-maker research skills  Policy-maker research awareness  Other | **Facilitators to use of evidence** contact with researchers/info staff  relationship with researchers/info staff  Relationship with policy-makers  Political support (will)  Political support (practical)  Format of research findings  Improved dissemination  Other | **Types of evidence used** Primary research  **Types of result** Perceptions  Intentions |
| Oxman (2007) (118)  **Study design** Qualitative study | **Time frame** Cross-sectional  **Data collection** Semi-structured interviews  Documentary analysis  **Data analysis** Thematic analysis | **Sample population** Policy-makers  **Setting** Policy environment  **Discipline** Health policy | **Barriers to use of evidence** Availability of research/access to research or info  Ambiguity/relevance/reliability of findings  Other |  | **Types of evidence used** Systematic reviews  Guidelines  Primary research  Other information - not formal research  **Types of result** Documentary proof of research use  Perceptions  Experiences |
| Percy-Smith (2002) (119)  **Study design** Case study  Qualitative study | **Time frame** Cross-sectional  **Data collection** Documentary analysis | **Sample population** Policy-makers  Local authority staff  **Setting** Policy environment  **Discipline** Other | **Barriers to use of evidence** Timing/opportunity  Managerial will  Political support (will)  Availability of research/access to research or info  Ambiguity/relevance/reliability of findings  Format of research findings  Other  importance of research findings | **Facilitators to use of evidence** Relationship with policy-makers  Timing/opportunity  Material resources available  Political support (will)  Managerial support (practical)  Availability of research/access to research or info  Clarity/relevance/reliability of research findings  Format of research findings  Policy-maker research awareness  professional bodies  Improved dissemination  Other  importance of policy | **Types of evidence used** Primary research  Other information - not formal research  **Types of result** Behaviour  Perceptions  Experiences |
| Petticrew (2004) (120)  **Study design** Qualitative study | **Time frame** Cross-sectional  **Data collection** Group interviews  **Data analysis** Thematic analysis | **Sample population** Policy advisor  **Setting** Other  **Discipline** public health | **Barriers to use of evidence** Timing/opportunity  Costs  Availability of research/access to research or info  Consumer-related barrier  Other | **Facilitators to use of evidence** contact with researchers/info staff  Relationship with policy-makers  Collaboration  Timing/opportunity  Availability of research/access to research or info  Clarity/relevance/reliability of research findings  Format of research findings  Other  importance of policy | **Types of evidence used** Primary research  Other information - not formal research  **Types of result** Behaviour  Perceptions  Experiences |
| Priest (2009) (121)  **Study design** Case study | **Time frame** Cross-sectional  **Data collection** not stated  **Data analysis** Not stated | **Sample population** Policy advisor  Policy-makers  **Setting** Policy environment  **Discipline** Health policy  public health |  | **Facilitators to use of evidence** Availability of research/access to research or info  Clarity/relevance/reliability of research findings  Format of research findings  Improved dissemination | **Types of evidence used** Primary research  **Types of result** Documentary proof of research use  suggestions/proposed factors  Perceptions |
| Qazi (2011) (122)  **Study design** Qualitative study | **Time frame** Cross-sectional  **Data collection** Semi-structured interviews  **Data analysis** Thematic analysis | **Sample population** Physicians  Health care managers  **Setting** Health care  **Discipline** Primary care  Secondary & tertiary care | **Barriers to use of evidence** Managerial support (practical)  Availability of research/access to research or info  Ambiguity/relevance/reliability of findings  Other | **Facilitators to use of evidence** Contact with policy makers  Timing/opportunity  Political support (will)  Managerial support (practical)  Availability of research/access to research or info  Clarity/relevance/reliability of research findings | **Types of evidence used** Other information - not formal research  **Types of result** Perceptions |
| Reed (2011) (123)  **Study design** Quantitative study | **Time frame** Cross-sectional  **Data collection** Survey  **Data analysis** Descriptive statistics | **Sample population** Researchers  Nurses  **Setting** Academic institution  **Discipline** Primary care  Health policy  Secondary & tertiary care |  | **Facilitators to use of evidence** importance of policy  importance of research findings | **Types of evidence used** Systematic reviews  Primary research  **Types of result** Perceptions |
| Rieckmann (2011) (124)  **Study design** Qualitative study | **Time frame** Cross-sectional  **Data collection** Semi-structured interviews  **Data analysis** Thematic analysis | **Sample population** Policy-makers  Health care managers  **Setting** Health care  **Discipline** drugs policy |  | **Facilitators to use of evidence** Other | **Types of evidence used** Not stated  **Types of result** Perceptions |
| Rigby (2005) (125)  **Study design** Qualitative study | **Time frame** Cross-sectional  **Data collection** not stated  **Data analysis** Not stated | **Sample population** Policy advisor  **Setting** Policy environment  **Discipline** Other | **Barriers to use of evidence** contact with researchers/info staff  Costs  Availability of research/access to research or info  Ambiguity/relevance/reliability of findings  Other  importance of policy | **Facilitators to use of evidence** contact with researchers/info staff  Contact with policy makers  relationship with researchers/info staff  Relationship with policy-makers  Collaboration  Timing/opportunity  Clarity/relevance/reliability of research findings  Format of research findings  professional bodies  Improved dissemination  Other | **Types of evidence used** Systematic reviews  Guidelines  Primary research  Other information - not formal research  **Types of result** Perceptions  Experiences |
| Ritter (2009) (126)  **Study design** Qualitative study | **Time frame** Cross-sectional  **Data collection** Semi-structured interviews  **Data analysis** Content analysis | **Sample population** Policy-makers  Other  **Setting** Health care  Policy environment  Other  **Discipline** drugs policy | **Barriers to use of evidence** Timing/opportunity  Availability of research/access to research or info | **Facilitators to use of evidence** contact with researchers/info staff  Contact with policy makers  relationship with researchers/info staff  Timing/opportunity  Availability of research/access to research or info  Clarity/relevance/reliability of research findings  Guidelines or policy statement  Other | **Types of evidence used** Systematic reviews  Guidelines  Primary research  Other information - not formal research  **Types of result** Perceptions  Experiences |
| Rocchi (2008) (127)  **Study design** Qualitative study | **Time frame** Cross-sectional  **Data collection** Focus Group  **Data analysis** Thematic analysis | **Sample population** Researchers  Policy advisor  Policy-makers  Health care managers  Other  **Setting** Other  **Discipline** drugs policy | **Barriers to use of evidence** Availability of research/access to research or info  Ambiguity/relevance/reliability of findings  Lack of legal support | **Facilitators to use of evidence** importance of policy | **Types of evidence used** Primary research  **Types of result** Perceptions |
| Sleet (2011) (128)  **Study design** Systematic review  Qualitative study | **Time frame** Longitudinal  **Data collection** OTher  **Data analysis** Not stated | **Sample population** Other  **Setting** Policy environment  **Discipline** transport |  | **Facilitators to use of evidence** Political support (will) | **Types of evidence used** Systematic reviews  **Types of result** Other |
| Smith (2012) (129)  **Study design** Case study | **Time frame** Longitudinal  **Data collection** Semi-structured interviews  **Data analysis** Thematic analysis | **Sample population** Researchers  Physicians  Policy advisor  Policy-makers  Other  **Setting** Health care  Policy environment  **Discipline** public health | **Barriers to use of evidence** contact with researchers/info staff  Contact with policy makers  Relationship with policy-makers | **Facilitators to use of evidence** Relationship with policy-makers  Staff or personnel resources  Policy-maker research awareness  Policy-maker research skills  Improved dissemination  Other  importance of research findings | **Types of evidence used** Systematic reviews  Primary research  Other information - not formal research  **Types of result** Perceptions |
| Stevens (2011) (130)  **Study design** Qualitative study | **Time frame** Cross-sectional  **Data collection** Semi-structured interviews  Observation - written notes  **Data analysis** Thematic analysis | **Sample population** Policy advisor  Policy-makers  **Setting** Policy environment  **Discipline** criminal justice |  | **Facilitators to use of evidence** relationship with researchers/info staff  Relationship with policy-makers  Political support (will) | **Types of evidence used** Systematic reviews  Guidelines  Primary research  Other information - not formal research  **Types of result** Perceptions |
| Stewart (2005)  (131)  **Study design** Qualitative study | **Time frame** Cross-sectional  **Data collection** Survey  Observation - written notes  **Data analysis** Not stated | **Sample population** Researchers  Nurses  Allied health professionals  Policy-makers  **Setting** Other  **Discipline** health promotion  public health | **Barriers to use of evidence** contact with researchers/info staff  Contact with policy makers  Timing/opportunity  Ambiguity/relevance/reliability of findings  Format of research findings | **Facilitators to use of evidence** Collaboration  Availability of research/access to research or info  Clarity/relevance/reliability of research findings  Practitioner research awareness  Practitioner research skills  Policy-maker research awareness  Policy-maker research skills | **Types of evidence used** Primary research  **Types of result** suggestions/proposed factors  Perceptions |
| Tran (2009) (132)  **Study design** Case study Qualitative study | **Time frame**  Cross-sectional  **Data collection** not stated  **Data analysis** Not stated | **Sample population** Researchers  Policy-makers  **Setting** Other  **Discipline** transport public health |  | **Facilitators to use of evidence** contact with researchers/info staff  Contact with policy makers  relationship with researchers/info staff  Relationship with policy-makers  Collaboration | **Types of evidence used** Not stated  **Types of result** Experiences |
| Tulloch (2011) (133)  **Study design** Case study | **Time frame** Cross-sectional  **Data collection** not stated  **Data analysis** Descriptive - no analysis | **Sample population** Researchers  Legal staff  **Setting** Health care **Setting** Policy environment  **Discipline** health promotion | **Barriers to use of evidence** importance of policy | **Facilitators to use of evidence** Relationship with policy-makers  Timing/opportunity  Political support (practical)  Managerial support (practical)  Availability of research/access to research or info  Clarity/relevance/reliability of research findings  Improved dissemination | **Types of evidence used** Not stated  **Types of result** Other |
| Uneke (2011) (134)  **Study design** Quantitative study | **Time frame** Cross-sectional  **Data collection** Survey  **Data analysis** Descriptive statistics | **Sample population** Researchers  Physicians  Policy advisor  Policy-makers  Health care managers  Other  **Setting** Health care  Policy environment  **Discipline** Health policy  public health | **Barriers to use of evidence** relationship with researchers/info staff  Costs  Lack of material resources  Availability of research/access to research or info  Ambiguity/relevance/reliability of findings  Format of research findings  Policy-maker research skills  Policy-maker research awareness  Other |  | **Types of evidence used** Not stated  **Types of result** Perceptions |
| Vingilis (2003) (135)  **Study design** Qualitative study | **Time frame** Cross-sectional  **Data collection** Documentary analysis  Observation - written notes  **Data analysis** Not stated | **Sample population** Researchers  Physicians  Policy advisor  Policy-makers  Health care managers  Other  **Setting** Academic institution  **Discipline** Other |  | **Facilitators to use of evidence** contact with researchers/info staff  Contact with policy makers  Collaboration | **Types of evidence used** Not stated  **Types of result** Experiences |
| von Lengerke (2004) (136)  **Study design** Quantitative study | **Time frame** Cross-sectional  **Data collection** Survey  **Data analysis** Descriptive statistics  Regression analysis | **Sample population** Policy-makers  **Setting** Policy environment  **Discipline** health promotion | **Barriers to use of evidence** Other | **Facilitators to use of evidence** Timing/opportunity  Political support (will) | **Types of evidence used** Not stated  **Types of result** Behaviour  Perceptions |
| Wang (2011) (137)  **Study design** Qualitative study | **Time frame** Cross-sectional  **Data collection** Survey  Semi-structured interviews  **Data analysis** Descriptive statistics | **Sample population** Policy-makers  Health care managers  **Setting** Health care  **Discipline** drugs policy | **Barriers to use of evidence** Managerial support (practical)  Availability of research/access to research or info  Ambiguity/relevance/reliability of findings  Other | **Facilitators to use of evidence** Availability of research/access to research or info  Clarity/relevance/reliability of research findings  Other | **Types of evidence used** Systematic reviews  Primary research  **Types of result** Perceptions |
| Ward (2012) (138)  **Study design** Case study | **Time frame** Cross-sectional  **Data collection** Semi-structured interviews **Data collection** Observation - written notes **Data collection** OTher  **Data analysis** Realist | **Sample population** Allied health professionals  **Setting** Health care  **Discipline** Primary care **Discipline** Secondary & tertiary care |  | **Facilitators to use of evidence** Other | **Types of evidence used** Not stated  **Types of result** Perceptions  Experiences |
| Weatherly (2002) (139)  **Study design** Quantitative study Qualitative study | **Time frame** Cross-sectional  **Data collection** Survey  Semi-structured interviews  Documentary analysis  **Data analysis** Thematic analysis  Descriptive statistics | **Sample population** Policy-makers  **Setting** Health care  **Discipline** Primary care  Secondary & tertiary care | **Barriers to use of evidence** Timing/opportunity  Staff or personnel resources  Availability of research/access to research or info  Ambiguity/relevance/reliability of findings  Policy-maker research skills  Policy-maker research awareness | **Facilitators to use of evidence** Guidelines or policy statement | **Types of evidence used** Systematic reviews  Guidelines  Primary research  Other information - not formal research  **Types of result** Perceptions  Experiences |
| Wehrens (2010) (140)  **Study design** Case study | **Time frame** Cross-sectional  **Data collection** Semi-structured interviews  Observation - written notes  **Data analysis** Thematic analysis | **Sample population** Researchers  Policy advisor  Policy-makers  **Setting** Policy environment  **Discipline** public health | **Barriers to use of evidence** Policy-maker research skills | **Facilitators to use of evidence** Collaboration  Other | **Types of evidence used** Not stated  **Types of result** Behaviour  Perceptions  Experiences |
| Wehrens (2011) (141)  **Study design** Case study Qualitative study | **Time frame** Cross-sectional  **Data collection** Semi-structured interviews  **Data analysis** Not stated | **Sample population** Researchers  Policy-makers  Health care managers  **Setting** Policy environment  **Discipline** Health policy | **Barriers to use of evidence** Availability of research/access to research or info  Ambiguity/relevance/reliability of findings | **Facilitators to use of evidence** contact with researchers/info staff  Contact with policy makers  relationship with researchers/info staff  Relationship with policy-makers  Collaboration  Timing/opportunity  opportunity/time to influence  Improved dissemination  Other  importance of policy | **Types of evidence used** Not stated  **Types of result** Perceptions  Experiences |
| Weitkamp (2012) (142)  **Study design** Qualitative study | **Time frame** Cross-sectional  **Data collection** Group interviews  **Data analysis** Descriptive statistics | **Sample population** Policy-makers  Local authority staff  **Setting** Policy environment  **Discipline** Conservation & environmental management |  | **Facilitators to use of evidence** Clarity/relevance/reliability of research findings  Format of research findings | **Types of evidence used** Other information - not formal research  **Types of result** Perceptions |
| Wilkins (2008) (143)  **Study design** other | **Time frame** Cross-sectional  **Data collection** Other  **Data analysis** Not stated | **Sample population** Physicians  Policy-makers  Health care managers  Local authority staff  **Setting** Policy environment  **Discipline** public health | **Barriers to use of evidence** Collaboration Timing/opportunity  Staff or personnel resources  Lack of material resources  Availability of research/access to research or info  Ambiguity/relevance/reliability of findings  Format of research findings  Policy-maker research skills  Policy-maker research awareness  Practitioner research awareness  Practitioner research skills  Other |  | **Types of evidence used** Other information - not formal research  **Types of result** Other |
| Williams (2008) (144)  **Study design** Systematic review Case study Qualitative study | **Time frame** Cross-sectional  **Data collection** Focus Group  Documentary analysis  Observation - written notes | **Sample population** Researchers  Physicians  Policy-makers  Other  **Setting** Health care  Policy environment  **Discipline** Primary care  Secondary & tertiary care | **Barriers to use of evidence** Collaboration  Timing/opportunity  Costs  Political support (will)  Lack of material resources  Availability of research/access to research or info  Ambiguity/relevance/reliability of findings  Policy-maker research skills  Other  Professional/international bodies | **Facilitators to use of evidence** Collaboration  Costs  Managerial support (practical)  Availability of research/access to research or info  Clarity/relevance/reliability of research findings  Format of research findings  Other | **Types of evidence used** Primary research  **Types of result** Behaviour  Experiences |
| Wuehler (2011) (145)  **Study design** Quantitative study other | **Time frame** Cross-sectional  **Data collection** Survey  Documentary analysis  **Data analysis** Not stated | **Sample population** Other  **Setting** Health care  Policy environment  **Discipline** health promotion Other | **Barriers to use of evidence** Availability of research/access to research or info  Ambiguity/relevance/reliability of findings |  | **Types of evidence used** Systematic reviews  Guidelines  Other information - not formal research  **Types of result** Documentary proof of research use  Perceptions |

Reference List

(1) Aaserud M, Lewin S, Innvaer S, Paulsen E, Dahlgren A, Trommald M, et al. Translating research into policy and practice in developing countries: a case study of magnesium sulphate for pre-eclampsia. BMC Health Services Research 2005;5(68):68.

(2) Albert MA, Fretheim A, Maiga D. Factors influencing the utilization of research findings by health policy-makers in a developing country: the selection of Mali's essential medicines. Health Research Policy and Systems 2007;5:2.

(3) Anaraki S, Plugge E. Delivering primary care in prison: The need to improve health information. Informatics in Primary Care 2003;11(4):191-4.

(4) Aoki-Suzuki C, Bengtsson M, Hotta Y. International Comparison and Suggestions for Capacity Development in Industrializing Countries. Journal of Industrial Ecology 2012 Aug;16(4):467-80.

(5) Armstrong R, Doyle J, Lamb C, Waters E. Multi-sectoral health promotion and public health: the role of evidence. Journal of Public Health 2006;28(2):168-72.

(6) Babu SC, Brown LR, McClafferty B. Systematic client consultation in development: the case of food policy research in Ghana, India, Kenya and Mali. World Development 2000 Jan;28(1):99-110.

(7) Baernholdt M, Lang NMMA. Government chief nursing officers' perceptions of barriers to using research on staffing. International Nursing Review 2007;54(1):49-55.

(8) Baghbanian A, Hughes I, Kebriaei A, Khavarpour FA. Adaptive decision-making: How Australian healthcare managers decide. Australian Health Review 2012;36(1):49-56.

(9) Barratt M. Organisational support for evidence-based practice within child and family social work: a collaborative study. Child and Family Social Work 2003;8(2):143-50.

(10) Bédard P, Ouimet M. Cognizance and Consultation of Randomized Controlled Trials among Ministerial Policy Analysts. Review of Policy Research 2012 Sep;29(5):625-44.

(11) Behague D, Tawiah C, Rosato M, Some T, Morrison J. Evidence-based policy-making: The implications of globally-applicable research for context-specific problem-solving in developing countries. Social Science & Medicine 2009 Nov;69(10):1539-46.

(12) Ben-Arieh A. The influence of social indicators data on decision making in regard to children's well-being. Administration in Social Work 2008;32(1):23-38.

(13) Best A, Greenhalgh T, Lewis S, Saul J, Carroll S, Bitz J. Large-System Transformation in Health Care: A Realist Review. Milbank Quarterly 2012 Sep;90(3):421-56.

(14) Bickford J, Kothari A. Research and knowledge in Ontario tobacco control networks. Canadian journal of public health Revue canadienne de sante publique 2008;99(4):297-300.

(15) Blackman T, Harrington B, Elliott E, Greene A, Hunter DJ, Marks L, et al. Framing health inequalities for local intervention: comparative case studies. Sociology of Health & Illness 2012 Jan;34(1):49-63.

(16) Blume S, Tump J. Evidence and policymaking: The introduction of MMR vaccine in the Netherlands. Social science & medicine (1982) 2010;71(6):1049-55.

(17) Boaz A, Hayden C. Pro-active evaluators: enabling research to be useful, usable and used. Evaluation 2002;8(4):440-53.

(18) Brambila C, Ottolenghi E, Marin C, Bertrand J. Getting results used: evidence from reproductive health programmatic research in Guatemala. Health Policy and Planning 2007;22(4):234-45.

(19) Brownson RC, Dodson EA, Stamatakis KA, Casey CM, Elliott MB, Luke DA, et al. Communicating evidence-based information on cancer prevention to state-level policy makers. Journal of the National Cancer Institute 2011;103(4):306-16.

(20) Bryce J, Victora C, Habicht J, Vaghan J, Black R. The Multi-Country Evaluation of the Integrated Management of Childhood Illness Strategy: Lessons for the Evaluation of Public Health Interventions. Am J Public Health 2004;20(Supp.1):94-105.

(21) Bunn F. Strategies to promote the impact of systematic reviews on healthcare policy: a systematic review of the literature. Evidence and Policy 2011;7:No-428.

(22) Bunn F, Kendall S. Does nursing research impact on policy? A case study of health visiting research and UK health policy. [References]. Journal of Research in Nursing 2011 Mar;Vol.16(2):169-91.

(23) Burris H, Parkhurst J, du-Sarkodie Y, Mayaud P. Getting research into policy - Herpes simplex virus type-2 (HSV-2) treatment and HIV infection: International guidelines formulation and the case of Ghana. Health Research Policy and Systems 2011;9(SUPPL. 1).

(24) Cameron A, Lart R, Salisbury C, Purdy S, Thorp H, Stewart K, et al. Policy makers' perceptions on the use of evidence from evaluations. Evidence and Policy 2011 Nov;7(4):429-48.

(25) Campbell DM, Redman S, Jorm L, Cooke M, Zwi A, Rychetnik L. Increasing the use of evidence in health policy: Practice and views of policy makers and researchers. Australia and New Zealand Health Policy 2006;6(21):not.

(26) Campbell D, Donald B, Moore G, Frew D. Evidence check: knowledge brokering to commission research reviews for policyAN - 857120904; 4175308. Evidence and Policy 2011 Jan;7(1):97-107.

(27) Carneiro M, Silva-Rosa T. The use of scientific knowledge in the decision making process of environmental public policies in Brazil. Journal of Science Communication 2011 Mar;10(1).

(28) Cerveny LK, Blahna DJ, Stern MJ, Mortimer MJ, Predmore SA, Freeman J. The use of recreation planning tools in U.S. Forest Service NEPA assessments. Environmental management 2011;48(3):644-57.

(29) Chambers D, Wilson P, Thompson C, Hanbury A, Farley K, Light K. Maximizing the Impact of Systematic Reviews in Health Care Decision Making: A Systematic Scoping Review of Knowledge-Translation Resources. Milbank Quarterly 2011 Mar;89(1):131-56.

(30) Chambers D, Wilson P, Grant R, Warren E, Pearson S. Use of evidence from systematic reviews to inform commissioning decisions: a case study. Evidence and Policy 2012 May;8(2):141-8.

(31) Coleman P. Influence of evidence-based guidance on health policy and clinical practice in England. Quality in Health Care 2001 Dec;10(4):229-37.

(32) Colon-Ramos U, Lindsay A, Monge-Rojas R, Greaney M, Campos H, Peterson K. Translating research into action: a case study on trans fatty acid research and nutrition policy in Costa Rica. Health policy and planning 2007;22(6):363-74.

(33) Contandriopoulos D, Lemire M, Denis J, Tremblay Ã. Knowledge Exchange Processes in Organizations and Policy Arenas: A Narrative Systematic Review of the Literature. Milbank Quarterly 2010 Dec;88(4):444-83.

(34) Currie L, Clancy L. The road to smoke-free legislation in Ireland. [References]. Addiction 2011 Jan;Vol.106(1):15-24.

(35) Deelstra Y, Nooteboom SG, Kohlmann HR, Berg J, Innanen S. Using knowledge for decision-making purposes in the context of large projects in The Netherlands. Environmental Impact Assessment Review 2003;23(5):517-41.

(36) Dionne F. Decision maker views on priority setting in the Vancouver Island Health Authority. 2006;not found(not found):not.

(37) Djibuti M, Rukhadze N, Hotchkiss D, Eisele T, Silvestre E. Health systems barriers to effective use of infectious disease surveillance data in the context of decentralization in Georgia: a qualitative study. Health Policy 2007;83(2-3):323-31.

(38) Dobbins M, Cockerill R, Barnsley J, Ciliska D. Factors of the innovation, organization, environment, and individual that predict the influence five systematic reviews had on public health decisions. International journal of technology assessment in health care 2001;17(4):467-78.

(39) Dobbins M, Jack S, Thomas H, Kothari A. Public health decision-makers' informational needs and preferences for receiving research evidence. 2007;4:156-63.

(40) Dobbins M, Robeson P, Ciliska D, Hanna S, Cameron R, O'Mara L, et al. A description of a knowledge broker role implemented as part of a randomized controlled trial evaluating three knowledge translation strategies. Implementation Science 2009;4(23).

(41) Dobrow MJ, Goel V, Lemieux-Charles L, Black NA. The impact of context on evidence utilization: a framework for expert groups developing health policy recommendations. Social science & medicine (1982) 2006;63(7):1811-24.

(42) Dodson EA, Eyler AA, Chalifour S, Wintrode CG. A review of obesity-themed policy briefs. American Journal of Preventive Medicine 2012;43(3 SUPPL.2):S143-S148.

(43) Eddama O. A systematic review of the use of economic evaluation in local decision-making. Health Policy 2008;86(2):129-41.

(44) Egmond Sv, Bekker M, Bal R. Connecting evidence and policy: bringing researchers and policy makers together for effective evidence-based health policy in the Netherlands: a case studyAN - 857121397; 4175304. Evidence and Policy 2011 Jan;7(1):25-40.

(45) El-Jardali F, Lavis JN, Ataya N, Jamal D. Use of health systems and policy research evidence in the health policymaking in eastern Mediterranean countries: views and practices of researchers. Implementation Science 2012;7:2.

(46) Elliott H, Popay J. How are policy makers using evidence? Models of research utilisation and local NHS policy making. Journal of Epidemiology and Community Health 2000;54:461-8.

(47) Elshaug AG, Hiller JE, Moss JR. Exploring policy-makers' perspectives on disinvestment from ineffective healthcare practices. International journal of technology assessment in health care 2008;24(1):1-9.

(48) Ensor T, Clapham S, Prasai DP. What drives health policy formulation: insights from the Nepal maternity incentive scheme? Health Policy 2009;90(2-3):247-53.

(49) Ettelt S, Mays N. Health services research in Europe and its use for informing policy. Journal of health services research & policy 2011;16 Suppl 2:48-60.

(50) Feldman PHN. Improving communication between researchers and policy makers in long-term care or "researchers are from Mars; policy makers are from Venus". Center for Home Care Policy & Research Policy Briefs 2001;(5):1-6.

(51) Fickel JJT. Policymaker use of quality of care information. International Journal for Quality in Health Care 2005 Dec;17(6):497-504.

(52) Figgs LWB. Uses of Behavioral Risk Factor Surveillance System data, 1993-1997. American Journal of Public Health 2000 May;90(5):774-6.

(53) Flitcroft K, Gillespie J, Salkeld G, Carter S, Trevena L. Getting evidence into policy: the need for deliberative strategies? Social Science and Medicine 2011 Apr;72(7):1039-46.

(54) Fournier MF. Knowledge mobilization in the context of health technology assessment: An exploratory case study. Health Research Policy and Systems 2012;10(1):1-13.

(55) Frey K, Widmer T. Revising Swiss Policies: The Influence of Efficiency Analyses. American Journal of Evaluation 2011 Dec;32(4):494-517.

(56) Friese B, Bogenschneider K. The Voice of Experience: How Social Scientists Communicate Family Research to Policymakers. Family Relations 2009;58(2):229-43.

(57) Gagliardi AR, Fraser N, Wright FC, Lemieux-Charles L, Davis D. Fostering knowledge exchange between researchers and decision-makers: exploring the effectiveness of a mixed-methods approach. Health Policy 2008;86(1):53-63.

(58) Galani C. Self-reported healthcare decision-makers' attitudes towards economic evaluations of medical technologies. Current Medical Research and Opinion 2008;24(11):3049-58.

(59) Garrib A, Stoops N, McKenzie A, Dlamini L, Govender T, Rohde J, et al. An evaluation of the District Health Information System in rural South Africa. South African medical journal = Suid-Afrikaanse tydskrif vir geneeskunde 2008;98(7):549-52.

(60) Gkeredakis E, Swan J, Powell J, Nicolini D, Scarbrough H, Roginski C, et al. Mind the gap: Understanding utilisation of evidence and policy in health care management practice. Journal of Health, Organisation and Management 2011;25(3):298-314.

(61) de Goede J. Public health knowledge utilisation by policy actors: an evaluation study in Midden-Holland, the Netherlands. Evidence and Policy 2011;7(1):January-24.

(62) Comptroller and Auditor General of the National Audit Office. Getting the Evidence: Using Research in Policy Making. London: Stationery Office; 2003.

(63) Green A, Gerein N, Mirzoev T, Bird P, Pearson S, Anh LV, et al. Health policy processes in maternal health: A comparison of Vietnam, India and China. [References]. Health Policy 2011 May;Vol.100(2-3):167-73.

(64) Greyson DL, Cunningham C, Morgan S. Information behaviour of Canadian pharmaceutical policy makers. Health information and libraries journal 2012;29(1):16-27.

(65) Hamel N, Schrecker T. Unpacking capacity to utilize research: A tale of the Burkina Faso Public Health Association. Social Science & Medicine 2011 Jan;72(1):31-8.

(66) Haynes AS, Gillespie JA, Derrick GE, Hall WD, Redman S, Chapman S, et al. Galvanizers, guides, champions, and shields: The many ways that policymakers use public health researchers. Milbank Quarterly 2011;89(4):564-98.

(67) Haynes AS, Derrick GE, Chapman S, Redman S, Hall WD, Gillespie J, et al. From "our world" to the "real world": Exploring the views and behaviour of policy-influential Australian public health researchers. Social Science & Medicine 2011 Apr;Vol.72(7):1047-55.

(68) Henderson CE, Young DW, Farrell J, Taxman FS. Associations among state and local organizational contexts: Use of evidence-based practices in the criminal justice system. Drug and alcohol dependence 2009;103 Suppl 1:S23-S32.

(69) Hennink MS. Using research to inform health policy: barriers and strategies in developing countries. [Review] [32 refs]. Journal of Health Communication 2005 Mar;10(2):163-80.

(70) Higgins JW, Strange K, Scarr J, Pennock M, Barr V, Yew A, et al. "It's a feel. That's what a lot of our evidence would consist of ": Public health practitioners' perspectives on evidence. Evaluation & the Health Professions 2011 Sep;Vol.34(3):278-96.

(71) Hinchcliff R, Poulos R, Ivers RQ, Senserrick T. Understanding novice driver policy agenda setting. Public Health 2011;125(4):217-21.

(72) Hinchcliff R, Ivers R, Poulos R, Senserrick T. Utilization of research in policymaking for graduated driver licensing. Am J Public Health 2010;100(11):2052-8.

(73) Hird JA. Policy Analysis for What? The Effectiveness of Nonpartisan Policy Research Organizations. Policy Studies Journal 2005;33(1):83-105.

(74) Hivon ML. Use of health technology assessment in decision making: coresponsibility of users and producers? International journal of technology assessment in health care 2005;21(2):268-75.

(75) Hobin EP, Riley B, Hayward S, Ruggiero ED, Birdsell J. Maximising the use of evidence: exploring the intersection between population health intervention research and knowledge translation from a Canadian perspectiveAN - 963635613; 4282720. Evidence and Policy 2012 Jan;8(1):97-115.

(76) Hooton N. Linking evidence with user voice for pro-poor policy: lessons from East Africa. Development in practice 2010 Nov;20(8):985-1000.

(77) Hunsmann M. Limits to evidence-based health policymaking: Policy hurdles to structural HIV prevention in Tanzania. Social science and medicine 2012;74(10):1477-85.

(78) Hutchinson E, Parkhurst J, Phiri S, Gibb DM, Chishinga N, Droti B, et al. National policy development for cotrimoxazole prophylaxis in Malawi, Uganda and Zambia: The relationship between Context, Evidence and Links. Health Research Policy and Systems 2011;9(SUPPL. 1).

(79) Hyder AA, Corluka A, Winch PJ, El-Shinnawy A, Ghassany H, Malekafzali H, et al. National policy-makers speak out: are researchers giving them what they need? Health Policy and Planning 2011;26(1):73-82.

(80) Innvaer S. The use of evidence in public governmental reports on health policy: an analysis of 17 Norwegian official reports (NOU). BMC Health Services Research 2009;9:177.

(81) Innvaer S, Vist G, Trommald M, Oxman A. Health policy-makers' perceptions of their use of evidence: a systematic review. Journal of Health Services & Research Policy 2002;7:239-44.

(82) Jack SM. Knowledge transfer and exchange processes for environmental health issues in Canadian Aboriginal communities. International Journal of Environmental Research and Public Health 2010;7(2):651-74.

(83) Jacobs R, Moran V. Uptake of mandatory outcome measures in mental health services. Psychiatrist 2010;34(8):338-43.

(84) Jbilou J, Amara N, Landry R. Research-based-decision-making in Canadian health organizations: a behavioural approach. Journal of medical systems 2007;31(3):185-96.

(85) Jenkins RAR. Bridging data and decision making: development of techniques for improving the HIV prevention community planning process. AIDS & Behavior 2005 Jun;9(2 Suppl):S41-S53.

(86) Jennings ET, Hall JL. Evidence-Based Practice and the Use of Information in State Agency Decision Making. Journal of Public Administration Research & Theory 2012 Apr;22(2):245-66.

(87) Jewell CJ, Bero LA. "Developing good taste in evidence": facilitators of and hindrances to evidence-informed health policymaking in state government. The Milbank Quarterly 2008;86(2):177-208.

(88) Jonsson K. Health systems research in Lao PDR: Capacity development for getting research into policy and practice. Health Research Policy and Systems 2007;5(11).

(89) Kapiriri L, Bondy SJ. Health practitioners' and health planners' information needs and seeking behavior for decision making in Uganda. International journal of medical informatics 2006;75(10-11):714-21.

(90) Kok MO, Vaandrager L, Bal R, Schuit J. Practitioner opinions on health promotion interventions that work: Opening the â€˜black boxâ€™ of a linear evidence-based approach. Social Science & Medicine 2012 Mar;74(5):715-23.

(91) Kok MO, Rodrigues A, Silva AP, de Haan S. The emergence and current performance of a health research system: lessons from Guinea Bissau. Health research policy and systems / BioMed Central 2012;10:5.

(92) Kothari A. "Interaction" and research utilisation in health policies and programs: Does it work? Health Policy 2005;71(1):117-25.

(93) Kothari A, Edwards N, Hamel N, Judd M. Is research working for you? Validating a tool to examine the capacity of health organizations to use research. Implementation Science 2009;4:46.

(94) Kurko T, Silvast A, Wahlroos H, Pietila K, Airaksinen M. Is pharmaceutical policy evidence-informed? A case of the deregulation process of nicotine replacement therapy products in Finland. Health Policy 2012;105(2-3):246-55.

(95) Lairumbi GM, Molyneux S, Snow RW, Marsh K, Peshu N, English M. Promoting the social value of research in Kenya: examining the practical aspects of collaborative partnerships using an ethical framework. Social science & medicine (1982) 2008;67(5):734-47.

(96) Larsen M, Gulis G, Pedersen KM. Use of evidence in local public health work in Denmark. International journal of public health 2012 Jun;Vol.57(3):477-83.

(97) Lavis JN.Farrant MS.Stoddart GL. Barriers to employment-related healthy public policy in Canada. Health Promotion International 2001 Mar;16(1):9-20.

(98) Learmonth AM. Utilizing research in practice and generating evidence from practice. Health Education Research 2000 Dec;15(6):743-56.

(99) Lemieux-Charles LM. Building interorganizational knowledge for evidence-based health system change. Health Care Management Review 2002;27(3):48-59.

(100) Lencucha R, Kothari AR, Hamel N. Extending collaborations for knowledge translation: lessons from the community-based participatory research literature. Evidence and Policy 2010;6(1):January-75.

(101) Lewig K. The role of research in child protection policy reform: a case study of South Australia. Evidence and Policy 2010;6:No-482.

(102) Lomas J, Brown ADMA. Research and advice giving: A functional view of evidence-informed policy advice in a Canadian ministry of health. Milbank Quarterly 2009;87(4):903-26.

(103) Martin G, Currie G, Lockett A. Prospects for knowledge exchange in health policy and management: institutional and epistemic boundaries. Journal of health services research & policy 2011;16(4):211-7.

(104) McAneney H. Translating evidence into practice: a shared priority in public health? Social science and medicine 2010;70(10):May-1500.

(105) McBride T, Coburn A, Mackinney C, Mueller K, Slifkin R, Wakefield M. Bridging health research and policy: effective dissemination strategies. Journal of public health management and practice 2008;14(2):150-4.

(106) McDavid JC, Huse I. Legislator uses of public performance reports: Findings from a five-year study. American Journal of Evaluation 2012 Mar;33(1):7-25.

(107) McLaughlin A. Decision-making and evidence in direct practice. Clinical Social Work Journal 2010;38(2):June-163.

(108) Mercer SL. Translating evidence into policy: Lessons learned from the case of lowering the legal blood alcohol limit for drivers. Annals of Epidemiology 2010 Jun;20(6):412-20.

(109) Mitton CP. Evidence-based priority-setting: what do the decision-makers think? Journal of Health Services & Research Policy 2004 Jul;9(3):146-52.

(110) Moore G, Redman S, Haines M, Todd A. What works to increase the use of research in population health policy and programmes: a review. Evidence and Policy 2011 Aug;7(3):277-306.

(111) Murthy L, Shepperd S, Clarke M, Garner S, Lavis J, Perrier L, et al. Interventions to improve the use of systematic reviews in decision-making by health system managers, policy makers and clinicians. Cochrance Database of Systematic Reviews 2012;12(9):CD009401.

(112) Niedzwiedzka BM. Barriers to evidence-based decision making among Polish healthcare managers. Health Services Management Research 2003 May;16(2):106-15.

(113) Norton WE. An exploratory study to examine intentions to adopt an evidence-based HIV linkage-to-care intervention among state health department AIDS directors in the United States. Implementation Science 2012;7:27.

(114) Olson B, Armstrong EP, Grizzle AJ, Nichter MA. Industry's perception of presenting pharmacoeconomic models to managed care organizations. Journal of Managed Care Pharmacy 2003 Mar;9(2):159-67.

(115) Orem JN, Mafigiri DK, Marchal B, Ssengooba F, Macq J, Criel B. Research, evidence and policymaking: the perspectives of policy actors on improving uptake of evidence in health policy development and implementation in Uganda. BMC public health 2012;12:109.

(116) Ortega-Argueta Aaoem, Baxter G, Hockings M. Compliance of Australian threatened species recovery plans with legislative requirements. Journal of Environmental Management 2011 Aug;92(8):2054-60.

(117) Orton L. The Use of Research Evidence in Public Health Decision Making Processes: Systematic Review. PLoS ONE 2011;6(7):e21704.

(118) Oxman AD, Lavis JN, Fretheim A. Use of evidence in WHO recommendations. Lancet 2007;369(9576):1883-9.

(119) Percy-Smith J, Burden T, Darlow A, Dawson L, Hawtin M, Ladi S. Promoting Change through Research: The impact of Research on Local Government. York: Joseph Rowntree Foundation, York Publishing Services; 2002.

(120) Petticrew M, Whitehead M, Macintyre SJ, Graham H, Egan M. Evidence for public health policy on inequalities: 1: The reality according to policymakers. Journal of Epidemiology and Community Health 2004;58(10):811-6.

(121) Priest N. Engaging policy makers in action on socially determined health inequities : developing evidence-informed cameos. Evidence and Policy 2009;5(1):53-70.

(122) Qazi MS, Ali M. Health management information system utilization in Pakistan: Challenges, pitfalls and the way forward. BioScience Trends 2011;5(6):245-54.

(123) Reed RL, Kalucy EC, Jackson-Bowers E, McIntyre E. What research impacts do Australian primary health care researchers expect and achieve? Health research policy and systems / BioMed Central 2011;9:40.

(124) Rieckmann TR, Kovas AE, Cassidy EF, McCarty D. Employing policy and purchasing levers to increase the use of evidence-based practices in community-based substance abuse treatment settings: Reports from single state authorities. [References]. Evaluation and Program Planning 2011 Nov;Vol.34(4):366-74.

(125) Rigby E. Linking research and policy on Capitol Hill: insights from research brokers. Evidence and Policy 2005;1(2):195-213.

(126) Ritter A. How do drug policy makers access research evidence? The International journal on drug policy 2009;20(1):70-5.

(127) Rocchi A, Menon D, Verma S, Miller E. The role of economic evidence in Canadian oncology reimbursement decision-making: to lambda and beyond. Value in health : the journal of the International Society for Pharmacoeconomics and Outcomes Research 2008;11(4):771-83.

(128) Sleet DA, Mercer SL, Cole KH, Shults RA, Elder RW, Nichols JL. Scientific evidence and policy change: Lowering the legal blood alcohol limit for drivers to 0.08% in the USA. Global Health Promotion 2011;18(1):23-6.

(129) Smith K, Joyce K. Capturing complex realities: understanding efforts to achieve evidence-based policy and practice in public health. Evidence and Policy 2012;8(1):January-78.

(130) Stevens A. Telling Policy Stories: An Ethnographic Study of the Use of Evidence in Policy-making in the UK. Journal of Social Policy 2011 Apr;40(2):237-55.

(131) Stewart R, Wiggins M, Thomas J, Oliver S, Brunton G, Ellison G. Exploring the evidence-practice gap: a workshop report on mixed and participatory training for HIV prevention in Southern Africa. Education for Health 2005 Jul;18(2):224-35.

(132) Tran NT, Hyder AA, Kulanthayan S, Singh S, Umar RSR. Engaging policy makers in road safety research in Malaysia: a theoretical and contextual analysis. Health Policy 2009;90(1):58-65.

(133) Tulloch O, Mayaud P, du-Sarkodie Y, Opoku BK, Lithur NO, Sickle E, et al. Using research to influence sexual and reproductive health practice and implementation in Sub-Saharan Africa: A case-study analysis. Health Research Policy and Systems 2011;9(SUPPL. 1).

(134) Uneke CJ, Ezeoha AE, Ndukwe CD, Oyibo PG, Onwe F, Igbinedion EB, et al. Individual and organisational capacity for evidence use in policy making in Nigeria: an exploratory study of the perceptions of Nigeria health policy makersAN - 897342221; 4238309. Evidence and Policy 2011 Aug;7(3):251-76.

(135) VIngilis E, Hartford K, Schrecker T, Mitchell B, Lent B, Bishop J. Integrating knowledge generation with knowledge diffusion and utilization: a case study analysis of the Consortium for Applied Research and Evaluation in Mental Health. Canadian Journal of Public Health 2003 Nov;94(6):468-71.

(136) von Lengerke TR. Research utilization and the impact of health promotion policy. Sozial- und Praventivmedizin 2004;49(3):185-97.

(137) Wang A, Baerwaldt T, Kuan R, Nordyke R, Halbert R. Payer perspectives on evidence for formulary decision making in the United States. Value in Health 2011;14(7):A350.

(138) Ward V, Smith S, House A, Hamer S. Exploring knowledge exchange: A useful framework for practice and policy. Social Science & Medicine 2012 Feb;74(3):297-304.

(139) Weatherly HD. Using evidence in the development of local health policies. Some evidence from the United Kingdom. [Review] [12 refs]. International journal of technology assessment in health care 2002;18(4):771-81.

(140) Wehrens R, Bekker M, Bal R. The construction of evidence-based local health policy through partnerships: Research infrastructure, process, and context in the Rotterdam 'Healthy in the City' programme. Journal of public health policy 2010;31(4):447-60.

(141) Wehrens R, Bekker M, Bal R. Coordination of research, policy and practice: a case study of collaboration in the field of public health. Science and public policy 2011 Dec;38(10):755-66.

(142) Weitkamp G, Van den Berg AE, Bregt AK, Van Lammeren RJA. Evaluation by policy makers of a procedure to describe perceived landscape openness. Journal of Environmental Management 2012;95(1):17-28.

(143) Wilkins K, Nsubuga P, Mendlein J, Mercer D, Pappaioanou M. The data for decision making project: assessment of surveillance systems in developing countries to improve access to public health information. Public health 2008;122(9):914-22.

(144) Williams I, McIver S, Moore D, Bryan S. The use of economic evaluations in NHS decision-making: a review and empirical investigation. Health technology assessment (Winchester, England) 2008;12(7):iii-175.

(145) Wuehler SE, Ouedraogo AW. Situational analysis of infant and young child nutrition policies and programmatic activities in Burkina Faso. Maternal and Child Nutrition 2011;7(SUPPL. 1):35-62.
